# Research on the anti-pull performance of a novel small-diameter steel tube-stud grouting connector

**DOI:** 10.1371/journal.pone.0353244

**Published:** 2026-07-22

**Authors:** Ming-su Zhou, En-he Bao, Cheng-jun Luo, Jian-fa Wang, Yong-song Wu, Chang-jian Xiao

**Affiliations:** 1 College of Civil Engineering, Guizhou University, Guiyang, China; 2 Guizhou Provincial Key Laboratory of Rock and Soil Mechanics and Engineering Safety, Guiyang, China; 3 International College, Krirk University, Bangkok, Thailand; Henan Polytechnic University, CHINA

## Abstract

To clarify the pull-out performance of steel tube-stud grouting (STG) connectors, this paper designed 18 test specimens, with main research parameters including embedment depth of stud (4d and 8d), steel type of stud, steel tube thickness, stud end diameter, and steel tube backing plate hole diameter. The study investigated the failure mode and deformation capacity of connectors, and strain patterns of the studs and steel tubes in the connectors, through pull-out tests and numerical simulation analysis. The study found a high degree of consistency between experimental and simulated results, and strain and stress in the studs gradually increased from the ends outward. This study fills an existing research gap regarding external-wall connections for steel structures and proposes an innovative, reliable connecting detail for prefabricated steel facade joints. Test results confirm that the proposed connection exhibits favourable pullout behaviour even with low-strength grout and a shallow stud embedment of 4d, which reduces material costs and simplifies field construction. The derived formula for yield load capacity achieves high prediction accuracy (maximum error is 11.3%) and applies to practical engineering calculations, thereby enhancing design efficiency and structural safety. In addition, this work provides theoretical underpinnings and experimental datasets for parametric optimization, engineering popularization, and the development of specifications for STG connectors.

## 1. Introduction

**Fig 1:**
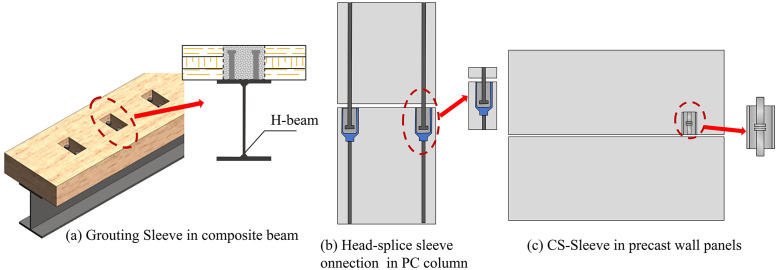
The engineering application of the sleeve connector.

**Fig 2:**
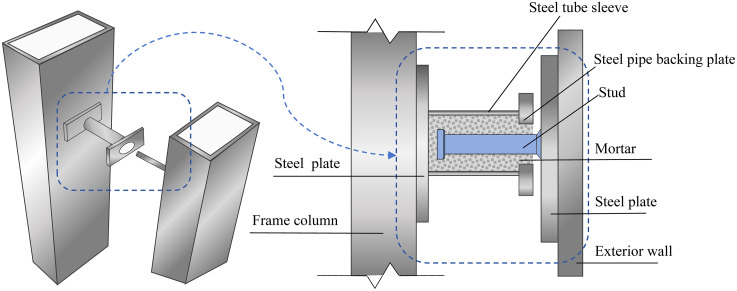
The position of the connector.

Inspired by the sleeve grouting connection technology, this paper proposes a new type of steel grouting tube connector, which is welded with perforated steel plates on both sides of the seamless steel tube, and used for the connection between the steel structure exterior wall and the main body. Compared to traditional sleeve grouting connectors, the STG connector proposed in this paper consists of low-strength mortar, a headed stud, and a steel tube. The engineering application of traditional sleeve connectors is illustrated in Fig 1. The detailed composition of the STG connector is shown in Fig 2.

Connection design plays a critical role in ensuring the overall performance of prefabricated structures [1]. The sleeve grouting connections, consisting of a sleeve, steel bars or studs, and grouting material, are widely used due to their versatility and cost-effectiveness [2]. Initially applied to connect precast concrete columns in frames, the technology has since been significantly improved. Sleeve grouting connections are commonly used in various prefabricated structure applications, including precast column-to-column and column-to-beam joints [3–7], offshore wind turbine support connections [8–10], vertical connections for prefabricated structure walls [11,12], and connections between columns and foundations, as well as between shear walls and foundations [13–17]. Additionally, many researchers have explored the performance and practical application of recycled concrete [18–22].

Many experimental studies have been conducted on sleeve grouting connectors. Ling [23–27] conducted a series of tensile tests on splicing sleeve connectors to examine their tensile capacity, failure modes, structural performance, bond strength, and ductility. Ling et al. also developed various new sleeve grouting connection joints. Alias et al. [28] performed direct tensile tests on 14 grouting sleeve connector specimens to evaluate their performance in precast concrete structures. The results showed that combining steel sleeves with steel spirals significantly improves the performance of connectors. Hosseini et al. [29] conducted pull-out tests on 36 grouted spiral connections and demonstrated that the pitch contributes more significantly to improving the bond performance than the diameter of spiral reinforcement. Johansen et al. [30] investigated the performance of grouting pile-sleeve connections in offshore jacket structures under alternating axial loads. The experiments showed that the cyclic load capacity of these sleeve connections is significantly lower than their static strength. Zhao et al. [31] established a formula for the bond strength of grouted sleeve connections using pull-out tests on 204 specimens and relevant literature data. On the basis of reliability analysis, they further put forward recommendations on the minimum anchorage length for various reinforcing bars. Grouted sleeve connections are characterized by superior tensile behavior, bond capacity, and ductility. Therefore, further research into grouted steel tube connections is highly necessary.

Numerous studies have also focused on the numerical simulation of sleeve grouted connections. Eliya et al. [32]conducted 3D finite element modelling on grouted connections with non-proprietary bar splicing sleeves, and determined the friction coefficients corresponding to failure loads and failure modes consistent with experimental results. Haber et al. [33] investigated the performance of two mechanical couplers in response to bridge design codes. This study proposed a simplified modelling method, which showed a strong agreement with experimental results, and it was easily implemented in software. Aragon et al. [34] elucidated the deformation mechanism of connections and the effect of sleeve parameters on mechanical performance via nonlinear finite element analysis, and further proposed recommended friction coefficients for interfacial slip of different types of grouted sleeves. Huang et al. [35] carried out static tests on 15 specimens to explore the tensile behavior of semi-grouted sleeve connections, and further established an analytical model for bond capacity along with corresponding design recommendations. AI-Jelawy [36] examined the monotonic tensile properties of grouting sleeves (GS) with different splicing configurations through experiments and numerical simulations. The results revealed that the failure modes of GS joints with various splicing configurations were consistent. Lv Na et al. [37] examined the effects of radial stiffness and shear key height-to-pitch ratio on the damage evolution and ultimate load capacity of grouted connections via experimental testing and finite element analysis. Previous studies have demonstrated the reliability of finite element analysis; therefore, this study also adopted this approach to facilitate an understanding of the behavior of STG.

**Fig 3:**
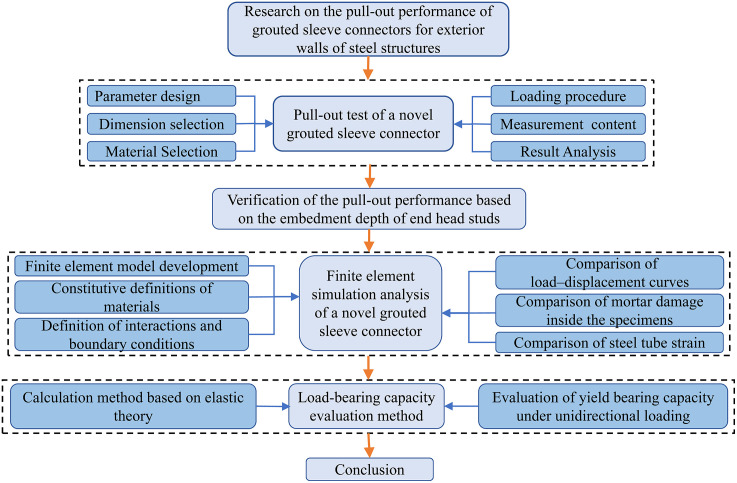
The flow chart of research technology.

The connectors between the exterior walls and the main structures rely on metal components, while the application of steel tube grouting connection technology has rarely been studied. To fill this gap, this paper proposes a novel grouted steel sleeve connection component. This connector exhibits prominent advantages, including high load-bearing capacity and excellent ductility, rendering it a promising connection node for exterior wall-main structure joints. To clarify the mechanical properties of the STG connectors, a comprehensive study was carried out via a combination of experimental tests, numerical simulations, and theoretical analysis, focusing on the effects of various parameters on the pull-out performance of the connectors. The main contributions and research significance of this paper are as follows: (1) The proposed connector, featuring low-cost, short embedment depth, and low-strength grout, can substantially cut material expenses, simplify construction procedures, improve site assembly efficiency, and satisfy the demands of industrialized construction. (2) Existing analytical theories for grouted connections primarily focus on rebar sleeve connections and lack dedicated mechanical models for the composite system consisting of steel tube confinement and stud-end bearing. In this work, a formula for yield bearing capacity is derived based on the Mohr–Coulomb strength criterion and elastic confinement mechanism, with calculation errors within 11.3%. This finding bridges the theoretical gap for steel-structural grouted connections and complements the analytical framework of such connection systems. (3) This study verifies that satisfactory pullout performance can be realized with medium–low strength grout, breaking the conventional design mindset of adopting high-grade grout and long embedment lengths. The research further clarifies the synergistic load-transfer mechanism among steel tube confinement, stud end bearing, and the localized grout crushing, supplying essential test data and theoretical foundations for future parametric optimization and numerical simulation. The research was conducted following a systematic technical route: experimental investigations were first performed on the components, followed by FE simulations and theoretical analyses based on the experimental findings. The detailed technical route is shown in Fig 3.

## 2. Experimental program

### 2.1. Specimen design

#### 2.1.1. Specimen configuration.

**Fig 4:**
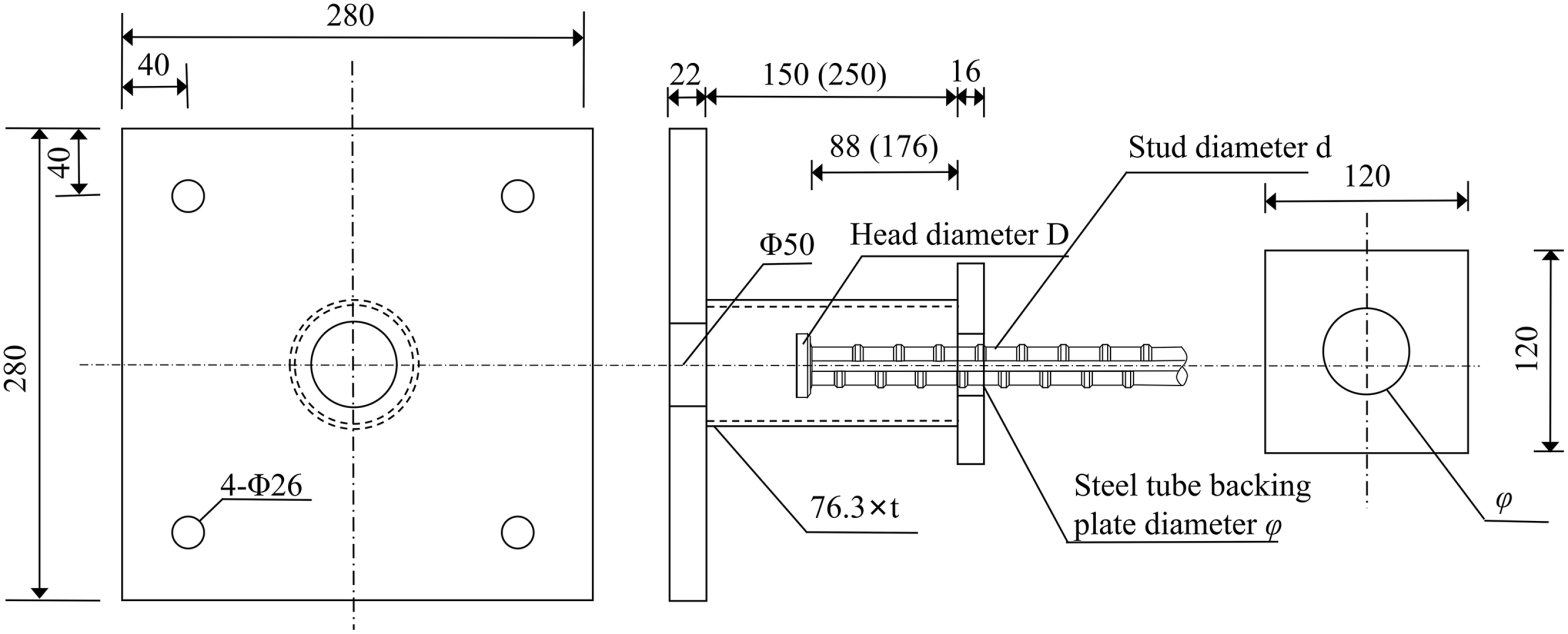
The geometric configuration diagram of the specimen.

The test adopted a small-diameter steel tube-stud grouting connector (STG connector), which consists of a seamless steel tube, a head-stud, a high-strength non-shrinkage grout, and a steel tube base plate. The steel tube has an inner diameter of 76.3 mm and two height specifications: 150 mm (stud embedment depth of 4d) and 250 mm (stud embedment depth of 8d). A 16 mm thick base plate (with grouting holes) and a 22 mm thick load-bearing steel plate are welded to the top and bottom ends of the steel tube, respectively. One head of the stud is welded with a circular end, and the stud is placed on the axial center of the steel tube. Grout is poured inside to form an integral load-bearing component. The specimen configuration is shown in Fig 4.

#### 2.1.2. Test parameters and grouping.

A total of 18 specimens, divided into 5 groups, were prepared for the test. The core variables included stud embedment depth, stud steel type, steel tube thickness, stud head diameter, and hole diameter in the base plate of the steel tube, all within typical engineering practice ranges. The stud diameter d was 22 mm. The stud embedment depths were 4d (88 mm) and 8d (176 mm). The stud steel types were deformed bars and plain round bars. Steel tube thicknesses were 2.8 mm and 4.2 mm. Stud head diameters were 35 mm and 44 mm. The diameters of the steel tube backing plate holes were 28 mm, 37 mm, and 46 mm. Detailed specimen parameters for each group are shown in Table 1.

**Table 1:** Parameters of each specimen.

Specimen number	Stud diameter d (mm)	Diameter of the steel tube (mm)	Embedment depth of stud (mm)	Head diameter D (mm)	Steel type of stud	Steel tube thickness t (mm)	Diameter of the steel tube backing plate *φ* (mm)
A1	22	76.3	88	35	Deformed stud	2.8	28
A2							37
A3				44			28
A4							37
A5							46
B1	22	76.3	176	35	Deformed stud	4.2	28
B2							37
B3				44			28
B4							37
B5							46
C1	22	76.3	88	35	Plain round stud	4.2	46
C2							37
C3						2.8	46
C4							37
D1	22	76.3	88	35	Deformed stud	4.2	46
D2						2.8	46
D3							37
E1	22	76.3	88	–	Deformed stud	4.2	37

### 2.2. Material property tests

#### 2.2.1. Mechanical properties of the studs.

**Fig 5:**
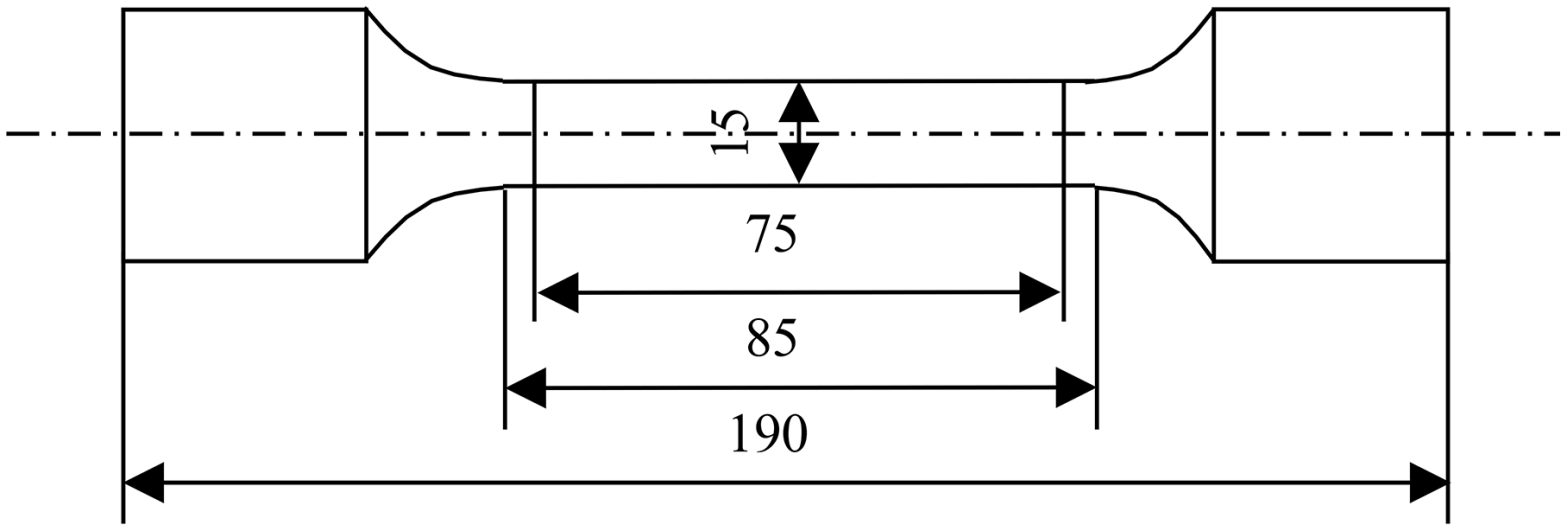
Schematic diagram of the specimen.

**Fig 6:**
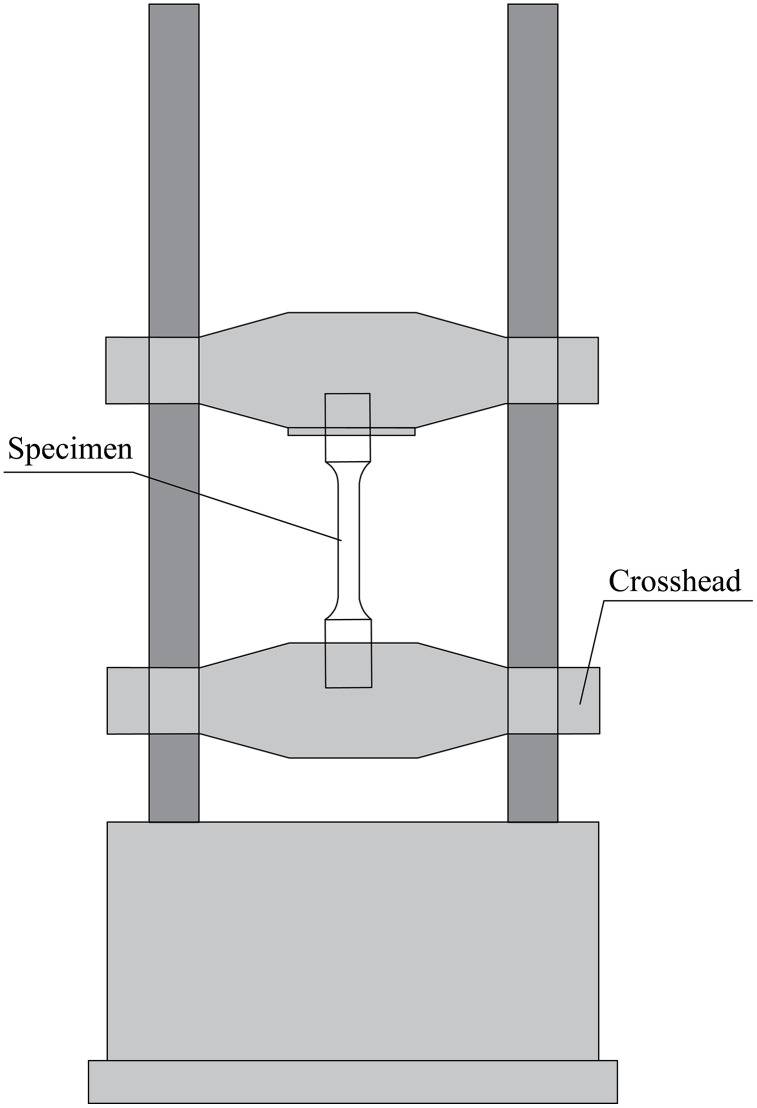
Loading diagram of the stud test.

were performed on a universal testing machine following GB/T 228.1–2021, *Metallic materials – Tensile testing – Part 1: Method of test at room temperature* [38]. Yield strength, ultimate tensile strength, percentage elongation after fracture, and elastic modulus were determined, and the measured data were averaged. The averaged results are listed in Table 2. The schematic diagram of the stud specimen and the loading diagram of the stud test are shown in Figs 5 and 6, respectively.

**Table 2:** Mechanical properties of studs.

Stud type	Yield strength *f*_*y*_/MPa	Tensile strength *f*_*u*_/MzPa	Elongation at break *A*/%	Elastic modulus *E*/MPa
Deformed stud	415.9	559.2	30.3	2.12 × 10^5^
Plain round stud	316.5	465.1	38.4	2.03 × 10^5^

#### 2.2.2. Mechanical properties of the steel tubes.

**Fig 7:**
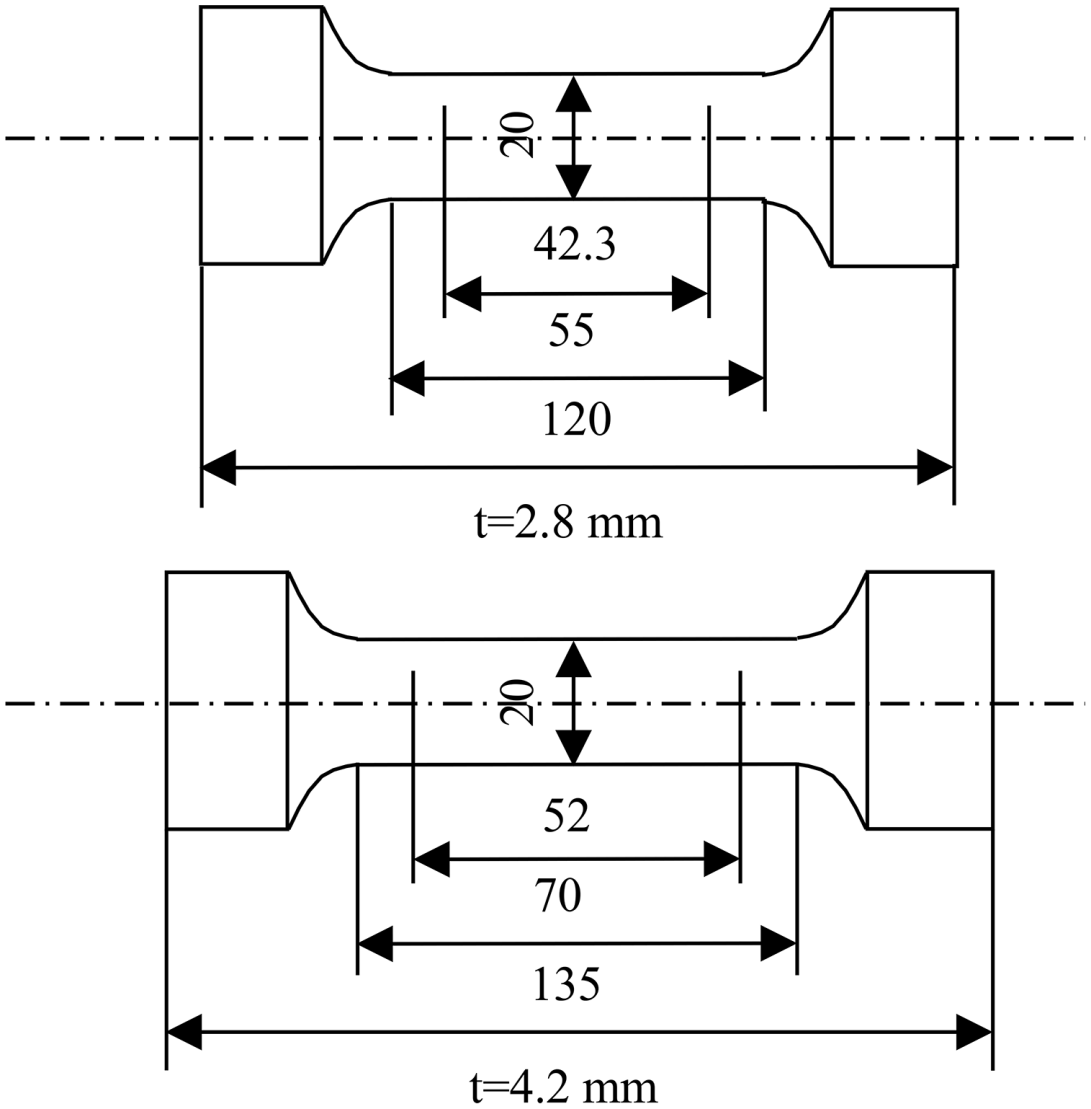
Schematic diagram of the specimen.

**Fig 8:**
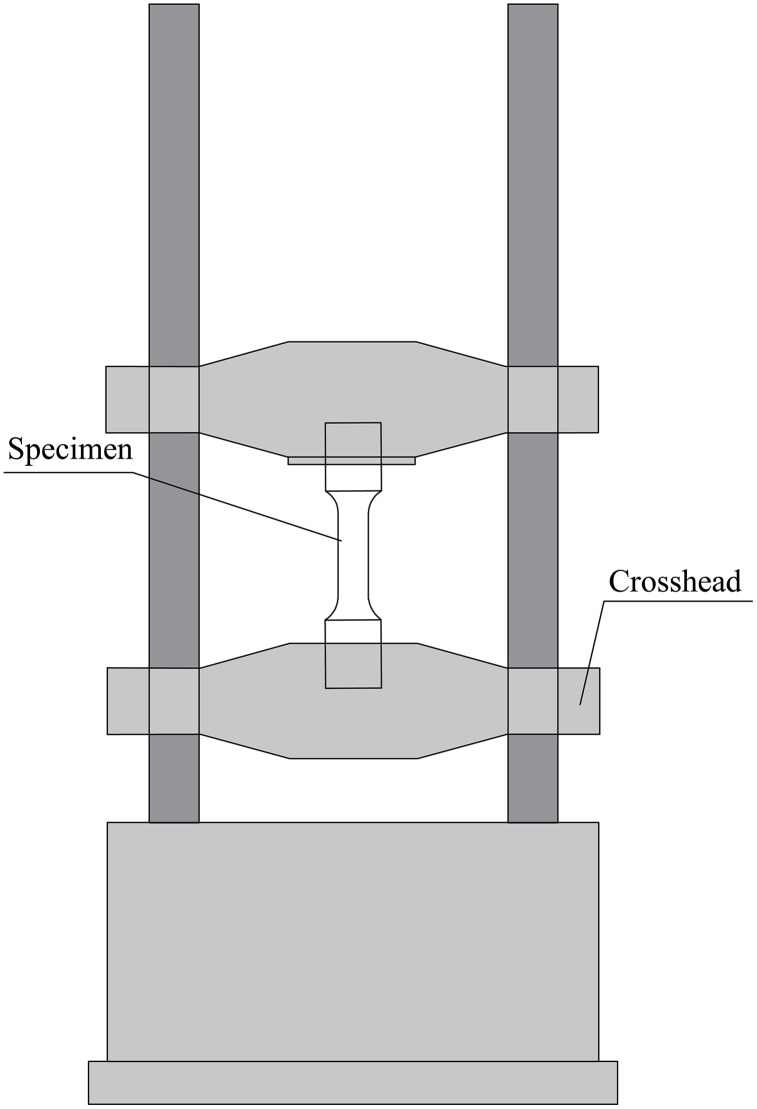
Loading diagram of the steel tube test.

Three longitudinal tensile coupons were sampled from each steel tube of the two wall thicknesses (2.8 mm and 4.2 mm). Uniaxial tensile tests following GB/T 228.1–2021 [38] were performed to characterize the mechanical properties of the parent steel, with test results summarized in Table 3. The schematic diagram of the tube specimen and the loading diagram of the tube test are shown in Figs 7 and 8, respectively.

**Table 3:** Mechanical properties of steel tubes.

Wall thickness of the steel tube	Yield strength *f*_*y*_/MPa	Tensile strength *f*_*u*_/MPa	Elongation at break *A*/%	Elastic modulus *E*/MPa
*t* = 4.2 mm	373.3	451.1	34.3	1.91 × 10^5^
*t* = 2.8 mm	332.8	426.6	36.8	1.79 × 10^5^

#### 2.2.3. Mechanical properties of the grout.

Cement-based grout was used as the filling material. Standard 40 mm × 40 mm × 160 mm prisms were prepared in compliance with GB/T 50448−2015 [39]. All grout prisms were cast alongside the main specimens and cured for 28 days and 44 days under the same conditions. Compression and splitting tensile tests were conducted to determine the compressive strength, splitting tensile strength, and elastic modulus, as listed in Table 4.

**Table 4:** Mechanical properties of grouting materials.

Grouting material group	Curing days (d)	Compressive strength (MPa)	Tensile strength (MPa)	Elastic modulus (MPa)
Group A	28	44.1	2.6	26900
Group B	28	44.7	2.6	28900
Group C	44	63.1	3.0	32200
Group D	44	63.5	2.9	32800
Group E	44	64.5	3.2	30900

### 2.3. Specimen fabrication process

**Fig 9:**
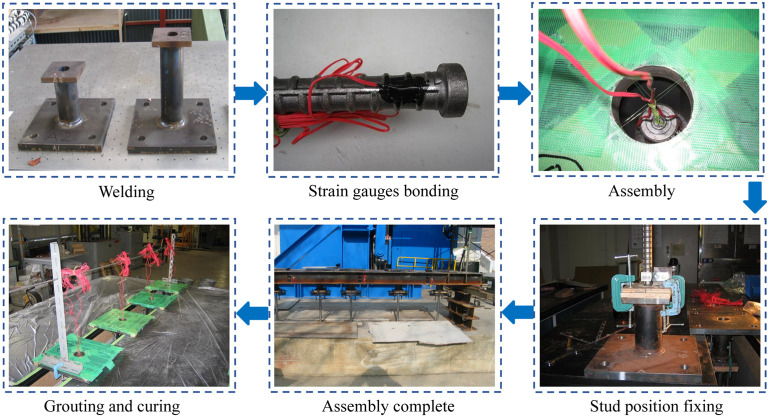
Specimen fabrication process.

The specimen fabrication process is illustrated in Fig 9. During specimen fabrication, the steel tubes were cut to the designed dimensions. The top and bottom base plates, along with the load-bearing steel plates, were welded to the tube ends to ensure welding quality and coaxiality. The strain gauges were symmetrically applied in two sets on the inner and outer walls of the steel tube, with one set on the studs aligned in the same plane as the tube. The studs were inserted into the steel tube to the designed embedment depth, ensuring the studs were coaxial with the tube. Multiple wooden boards were then secured to the steel tube backing plates using C-clamps. For grouting, the assembled specimens were filled until the grouting material was level with the grouting port. Grouting was deemed complete when no further settlement of the grout was observed. Standard test blocks were prepared and cured under the same environmental conditions as the specimens.

### 2.4. Loading setup and measurement scheme

#### 2.4.1. Loading setup and protocol.

**Fig 10:**
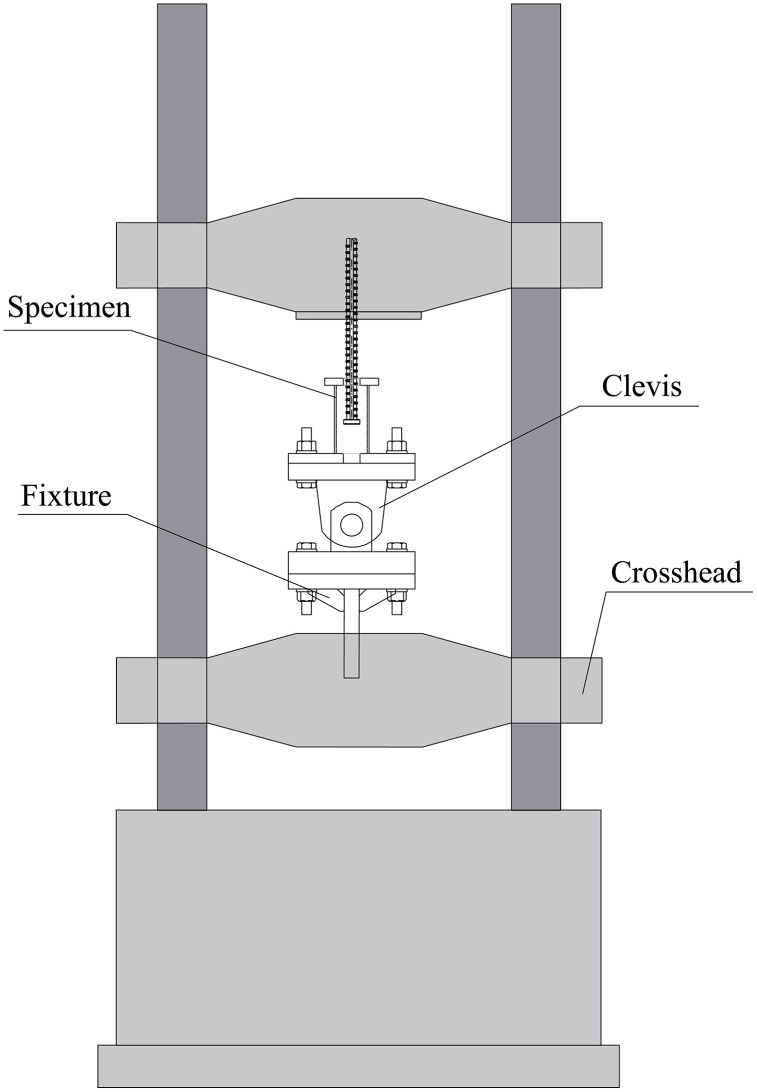
Schematic diagram.

**Fig 11:**
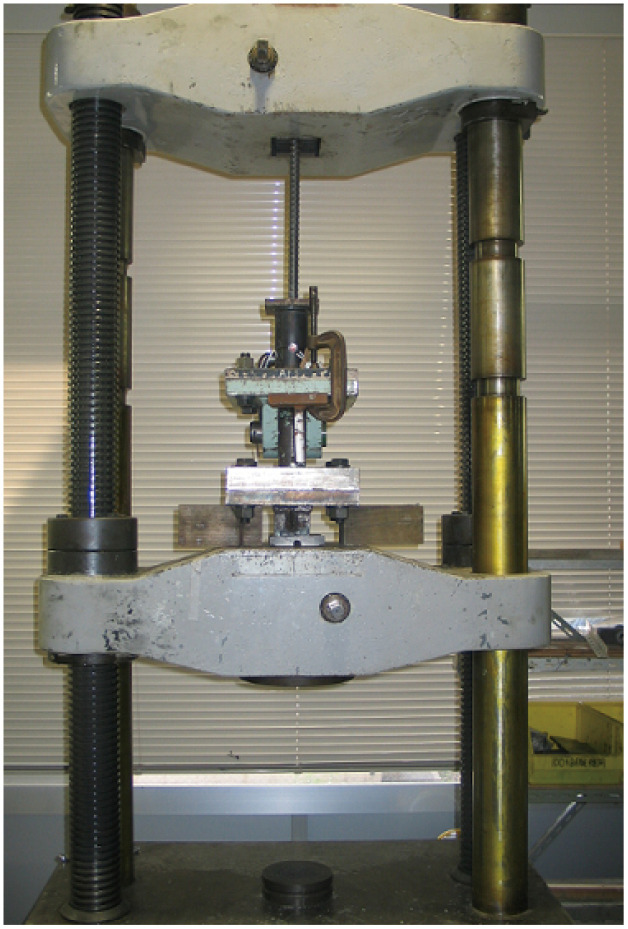
Photograph of actual equipment.

The monotonic pullout test was conducted using a 100 Tf universal testing machine, with loads applied concentrically along the stud axis. A schematic diagram of the test setup is shown in Fig 10, and the actual loading equipment is shown in Fig 11.

Loading was performed using a load-controlled, stepwise protocol with a constant loading rate for each step. Before formal loading, three cycles of incremental loading-unloading were carried out at load levels of 1/4, 1/2, and 3/4 of the stud’s theoretical yield load to eliminate assembly clearances and verify instrument readings. Thereafter, continuous constant-rate loading was applied until stud fracture or excessive pullout displacement occurred, at which point the test was terminated. Load, displacement, and strain were recorded in real time throughout the loading process.

#### 2.4.2. Measurement scheme.

**Fig 12:**
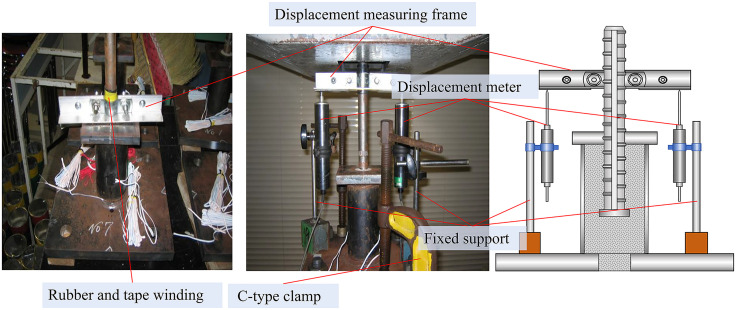
Installation of the displacement meters.

Fig 12 shows that the displacement meters were installed at symmetric positions on both the left and right sides. The distance between the displacement measuring frame and the steel tube backing plate was twice the diameter of the stud. The vertical displacement between the measuring frame and the lower steel plate of the steel tube was recorded as the total displacement. The average from the two displacement meters was used as the final displacement value.

**Fig 13:**
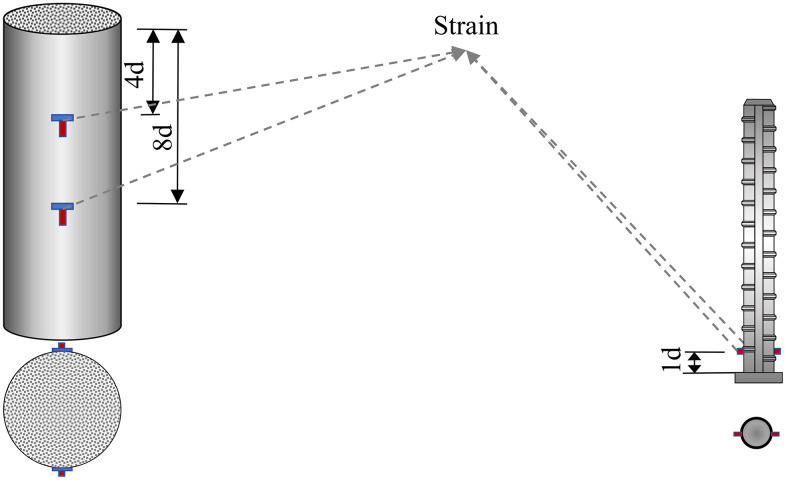
Strain gauge bonding locations.

The installation positions of the strain gauges are shown in Fig 13. Biaxial strain gauges were used to measure both axial and lateral strains of the steel tube. For groups A, C, D, and E, the measurement positions were located at the embedded depth of the studs, 4d from the upper end of the steel tube. For group B, the measurement positions were at 4d and 8d from the upper end. Besides, Axial strains at position 1d from the nut on the studs of specimens C1, C3, D1, and D2 were measured using uniaxial strain gauges.

## 3. Test results and analysis

### 3.1. Test results of the specimens

The initial stiffness *K*, yield load *P*_*y*_, ultimate load *P*_*u*_, maximum deformation *Δ*_max_ (defined as the deformation corresponding to ultimate load *P*_*u*_), and failure mode of each test specimen, as obtained from the pull-out tests, are summarized in Table 5. The initial stiffness is defined as the stiffness at the load point when the specimen reaches one-third of its maximum load. The yield bearing capacity corresponds to the load at which the specimen’s residual strain equals 0.2%.

**Table 5:** Main test results of specimens.

Specimen number	Initial stiffness *K*/(kN/mm)	Yield load *P*_*y*_/(kN)	Ultimate load *P*_*u*_/(kN)	Maximum deformation *Δ*_max_/(mm)	Failure mode
A1	432	147	200.91	15.76	Stud fracture
A2	504	148	200.64	19.30	
A3	386	151	204.53	13.87	
A4	476	155	207.71	14.53	
A5	380	154	207.93	14.41	
B1	473	152	206.70	15.04	Stud fracture
B2	373	154	205.25	22.20	
B3	417	154	208.02	13.77	
B4	399	154	208.75	15.52	
B5	395	155	207.31	14.39	
C1	273	115	175.74	39.33	Stud fracture
C2	286	117	176.92	55.31	
C3	280	113	174.25	25.60	
C4	330	113	175.67	24.12	
D1	324	146	200.06	21.34	Stud fracture
D2	312	148	204.88	30.73	
D3	329	149	205.15	20.79	
E1	466	149	189.91	7.46	Stud pull-out

Failure modes are categorised into two types: grouting material failure and stud fracture. In this test program, all specimens except E1 primarily exhibited stud shaft fracture, whereas the stud in specimen E1 underwent pull-out failure. The compressive stress *σ*_*c*_ (*σ*_*c*_ = *P*_*y*_/*A*_s_) of the grouting material was calculated using the bearing area *A*_s_ of the steel tube backing plate of each specimen and the yield load *P*_*y*_ of the stud, with values ranging from 60 to 80 N/mm^2^. Owing to the confinement effect of the steel tube and its backing plate, the strength of the grouting material was enhanced to a level sufficient to induce stud fracture, while the maximum bearing capacity exceeded the yield load of the stud. Therefore, the grouting material adopted in this experiment exhibits sufficient strength to fully exploit the mechanical performance of the studs.

### 3.2. Load-deformation curve

**Fig 14:**
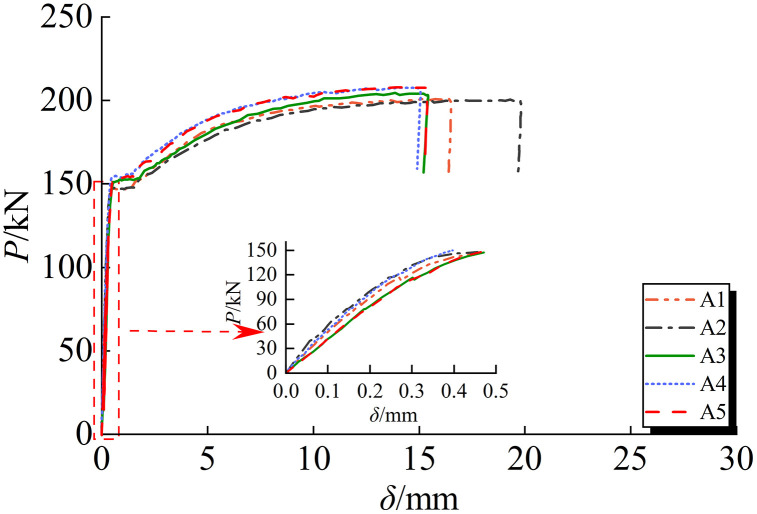
Load-deformation curves of GroupA.

**Fig 15:**
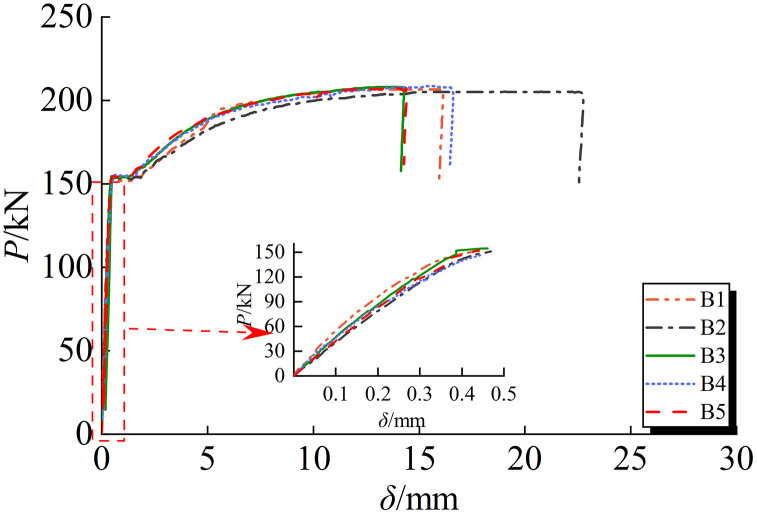
Load-deformation curves of Group B.

**Fig 16:**
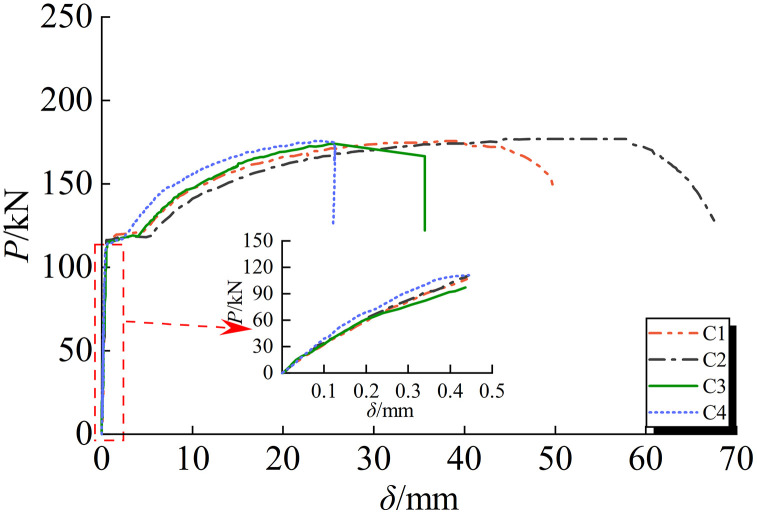
Load-deformation curves of Group C.

**Fig 17:**
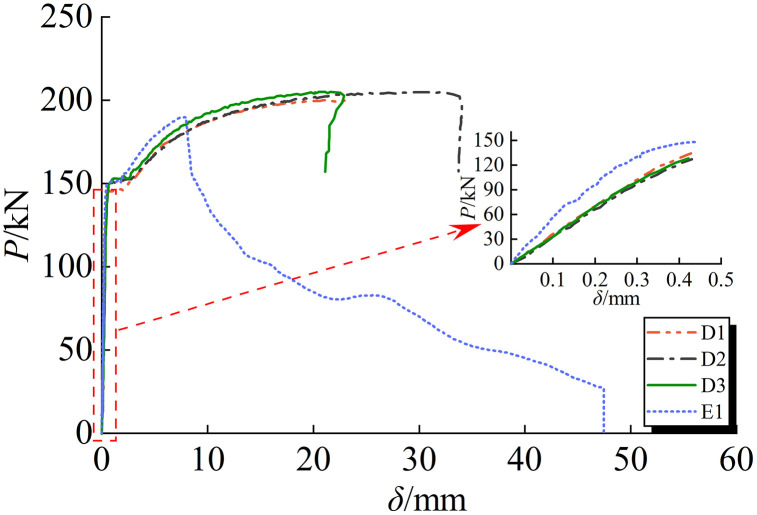
Load-deformation curves of Groups D and E.

Figs 14–17 illustrate the load-deformation curves for each specimen group. The vertical axis represents the applied load, while the horizontal axis represents the average displacement measured by the displacement meters mounted on both sides of the steel tube.

By observing the experimental process and referring to load-deformation curves, it can be seen that some specimens were unable to sustain the load after reaching their maximum load, with an instantaneous load drop occurring thereafter. This phenomenon is attributed to the error caused by the descent of the measurement frame after stud necking failure or grouting material damage in the specimens. If the necking position of the studs in the specimens was located between the displacement meter frame and the opening of the steel tube backing plate, the deformation exhibited an increasing trend, as exemplified by specimen C2 in Fig 16.

Based on the analysis of Figs 14–17, for groups A (stud embedment depth of 4d), B (stud embedment depth of 8d), and D (stud embedment depth of 4d), the stud heads in groups A and D were buried at a shallower depth, and the proportion of axial tensile force borne by their heads may be higher. Despite the differences in embedment depth, all three groups exhibited comparable strength and deformation capacity. Therefore, the stud embedment depth can be effectively reduced by machining the end structure of the deformed studs. For the group C specimens (smooth round steel studs), owing to the influence of the material properties of the stud steel, the bearing capacity of the specimens was relatively low, whereas their deformation performance was favorable. The stud of specimen E1 experienced pull-out failure, and its mechanical properties before yielding were consistent with those of the other specimens. It can thus be inferred that the stud head configuration exerts a negligible effect on the yield bearing capacity of the specimens. However, the ultimate deformation was relatively small, a phenomenon attributed to the absence of the optimized stud head.

Based on the above analysis, it can be concluded that as the hole diameter of the steel tube backing plate increases and the steel tube thickness decreases, the stiffness of the specimens tends to decrease, with no significant difference in overall mechanical performance observed. In addition, all specimens with stud heads failed due to stud fracture, demonstrating favorable load-bearing capacity and deformation performance. Therefore, it is confirmed that a steel tube thickness of 2.8 mm (with a diameter-to-thickness ratio of 27), a backing plate hole diameter of 46 mm (with a hole area ratio of 0.36, defined as the ratio of the backing plate hole area to the steel tube cross-sectional area), and a stud embedment depth of 4d can satisfy the service and load-bearing requirements.

### 3.3. Load-strain curves of the steel tube

**Fig 18:**
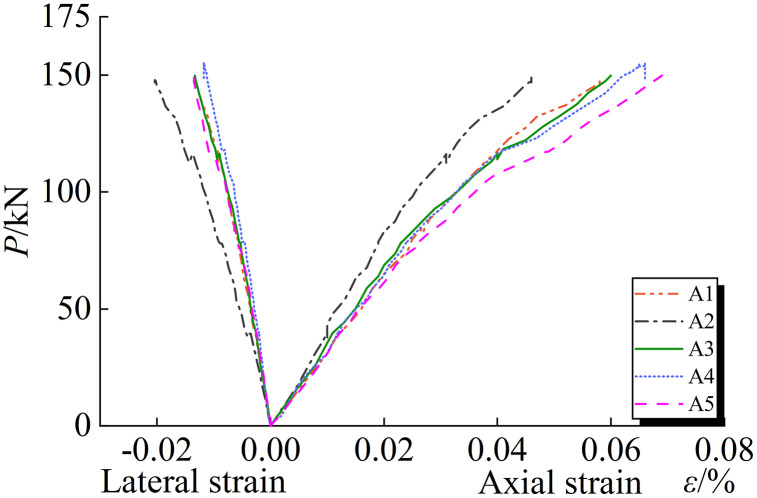
Load-strain curves of Group A.

**Fig 19:**
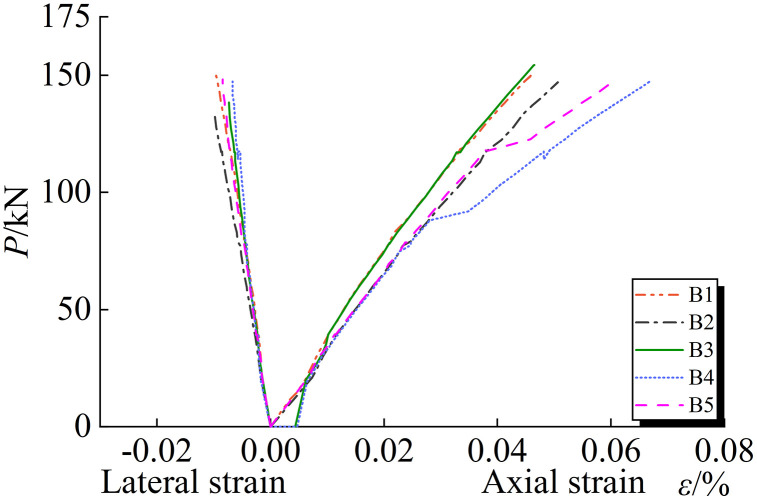
Load-strain curves of Group B (8d).

**Fig 20:**
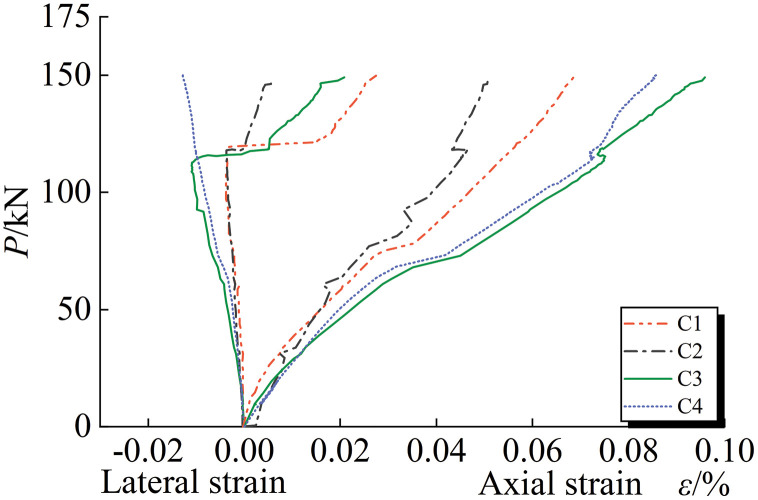
Load-strain curves of Group C.

**Fig 21:**
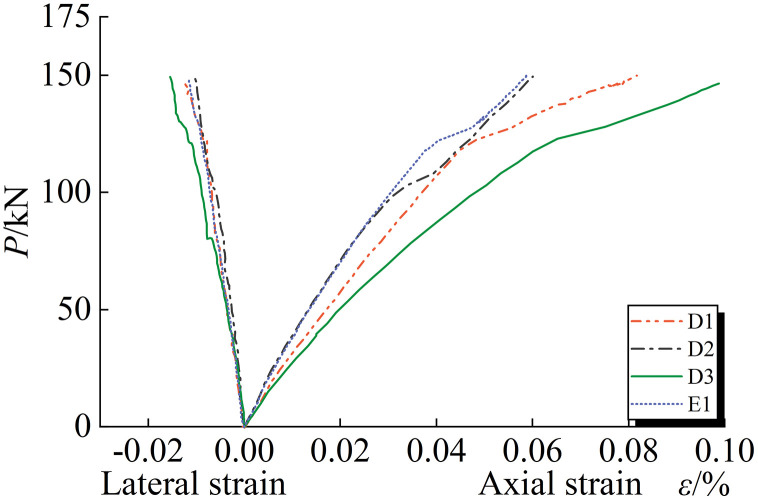
Load-strain curves of Groups D and E.

The load-strain curves of the steel tube are depicted in Fig 18–21. The vertical axis represents the load, and the horizontal axis shows the average strain values on both sides of the steel tube.

In Fig 18, all specimens except specimen A2 exhibited approximately identical response patterns. The fracture section of specimen A2 was located outside the steel tube, resulting in a smaller axial strain compared with that of the other specimens. Meanwhile, the slight damage to the grouting material inside the steel tube enhanced the interaction between the steel tube and grouting material during loading, leading to a larger lateral strain than observed in the other specimens. In Fig 19, specimen B4 was characterized by a relatively large stud head configuration and a moderate hole diameter of the steel tube backing plate. This specimen primarily sustained axial tensile force, with a relatively small transverse lateral pressure induced. In comparison with other specimens in group B, specimen B4 exhibited the maximum axial strain and the minimum lateral strain. Moreover, the axial and lateral strains of all specimens in group B followed essentially identical variation trends. In Fig 20, for specimen pairs with the same steel tube thickness (i.e., C1/C2 and C3/C4), the axial and lateral strain variation trends were similar within each pair. The axial strain of the group C specimens increased significantly at a load level of approximately 75 kN, and the lateral strain rose sharply as the load approached the yield point. This is because the studs are not provided with transverse ribs, which prevents them from forming a substantial mechanical interlock with the grouting material. Consequently, the stud heads engaged in advance, leading to more significant strain variations in the steel tube sections adjacent to the ends. The lateral strain variation trend of specimen C4 deviated from that of the other group C specimens, which is attributed to the shallower embedment depth of its studs. Under tensile loading, this specimen underwent premature yield failure, thus precluding a significant abrupt change in the steel tube strain. In Fig 21, given that both the D and E series specimens exhibited similar deformation characteristics, the influence of stud end configuration (presence or absence) and variations in grouting material strength on deformation can be ruled out. For Specimens D1 and D2, D1, with a thicker steel tube wall, exhibited greater axial and lateral deformation than D2. Specimen D3, which was characterized by the thinnest steel tube wall and the smallest steel tube backing plate hole diameter, demonstrated greater axial and lateral deformation than all other specimens in the series.

**Fig 22:**
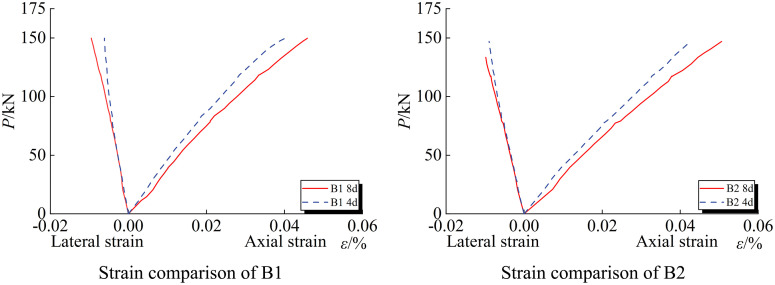
Strain comparison of different embedment depths for group B.

Fig 22 presents the load–strain curves of the steel cylinders at varying embedment depths (4d and 8d) for specimens B1 and B2. The schematic diagram of steel tube strain measurement is shown in Fig 12.

As shown in Fig 22, within the low-load range, the influence of stud embedment depth on lateral strain is negligible, with the initial lateral stiffness remaining essentially consistent. The greater the stud embedment depth, the higher the axial strain of the steel tube and the lower the corresponding axial stiffness. A comparison between specimens B1 and B2 indicates that, with all other parameters held constant, the larger the hole diameter of the steel tube backing plate, the higher the axial strain of the steel tube.

### 3.4. Axial load-strain curves of studs

**Fig 23:**
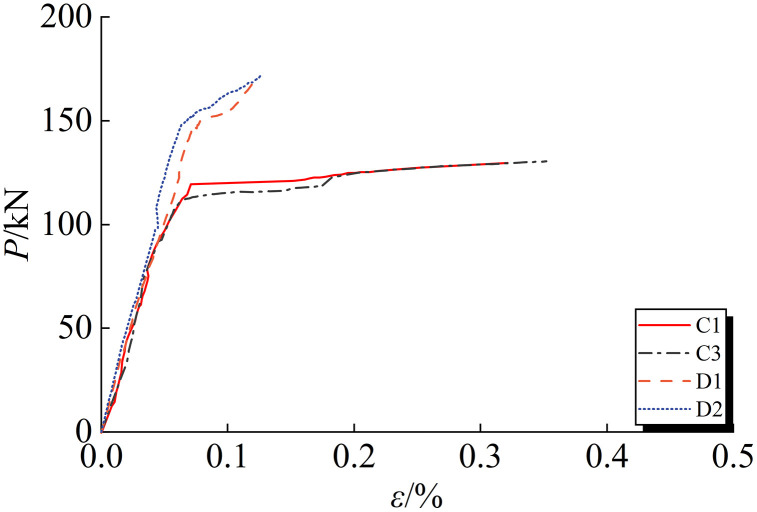
Axial load-strain curves of studs.

Fig 23 presents the axial load–strain curves of the studs. The vertical axis denotes the load, while the horizontal axis represents the average strain measured 1d from the stud heads on both sides of each stud, corresponding to the position shown in Fig 13.

As shown in Fig 23, the group C and D specimens feature identical stud diameters, thus exhibiting similar initial stiffness. Owing to the different steel types of the studs, the plastic-stage strain of the studs in specimens C1 and C3 is considerably higher than that in specimens D1 and D2, whereas the bearing capacity of the studs in specimens D1 and D2 is significantly greater than that in specimens C1 and C3.

### 3.5. Damage condition of the mortar at the steel tube sealing end

**Fig 24:**
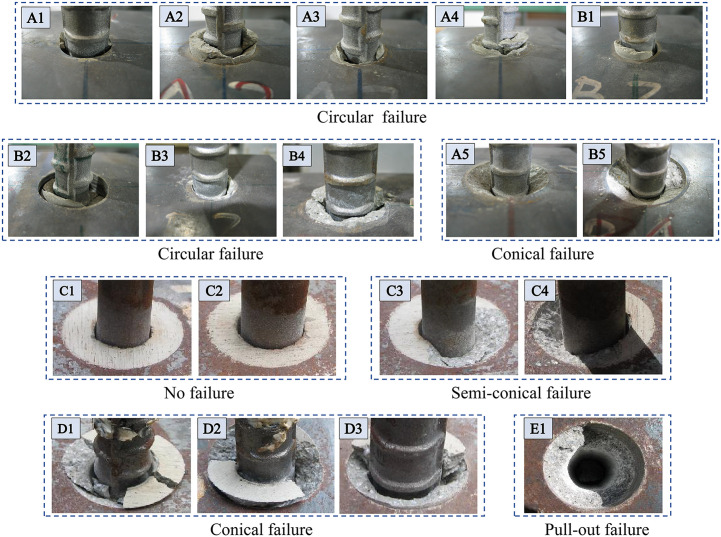
Mortar failure at the hole of the steel tube backing plate.

**Fig 25:**
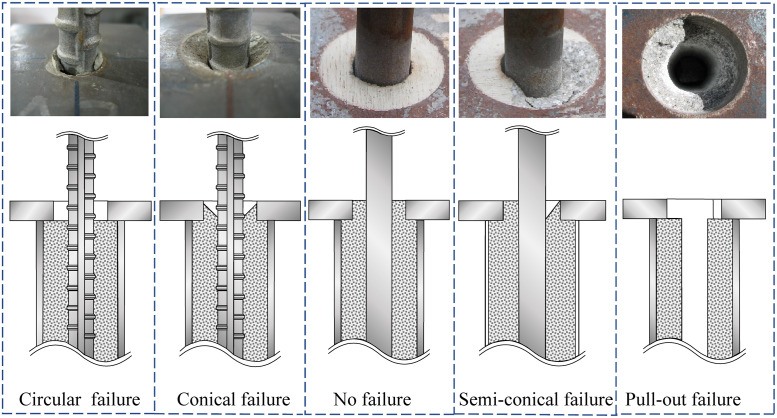
Schematic diagram of mortar failure at the steel tube backing plate.

Fig 24 shows the mortar failure at the hole diameter of the steel tube backing plate, and Fig 25 also provides the corresponding schematic.

Based on Figs 24 and 25, in groups A and B, the mortar at the steel tube backing plate of the specimen with a smaller hole diameter was drawn out along with the stud, resulting in circular failure, as shown in the leftmost panel of Fig 25. In contrast, larger hole diameters led to conical failure, as shown in the second panel from the left in Fig 25. It can be inferred that the larger the hole diameter, the greater the axial force sustained by the mortar at the hole of the steel tube backing plate.

For group C specimens, two failure modes were observed. Specimens C1 and C2 loosened without damaging the mortar at the hole of the steel tube backing plate, as shown in the third panel from the left in Fig 25. Meanwhile, specimens C3 and C4 exhibited both stud loosening and semi-conical mortar failure, as shown in the fourth panel from the left in Fig 25. The steel tube thickness for C1 and C2 was 4.2 mm, and for C3 and C4 was 2.8 mm. It can be inferred that in group C specimens, the thinner the tube wall, the greater the axial force sustained by the mortar at the hole diameter of the steel tube backing plate.

All group D specimens exhibited conical failure. Specimen E1 was pulled out entirely due to the absence of a stud end, resulting in the failure mode shown in the rightmost panel of Fig 25.

### 3.6. Mortar failure inside the steel tube

**Fig 26:**
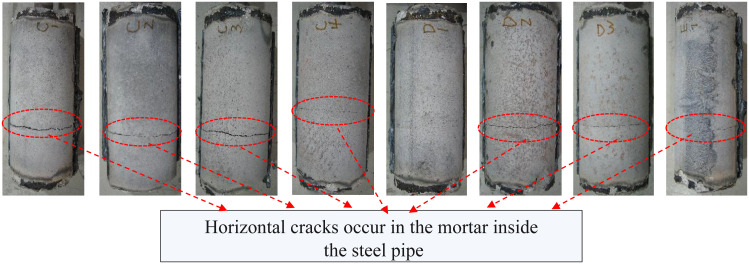
Cracks in mortar inside steel tubes of Groups C, D, and E.

**Fig 27:**
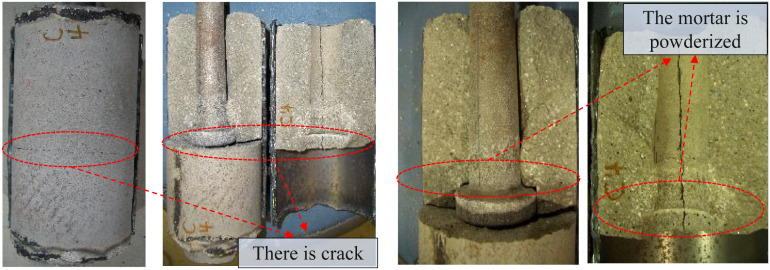
Mortar failure at the stud of specimen C4.

**Fig 28:**
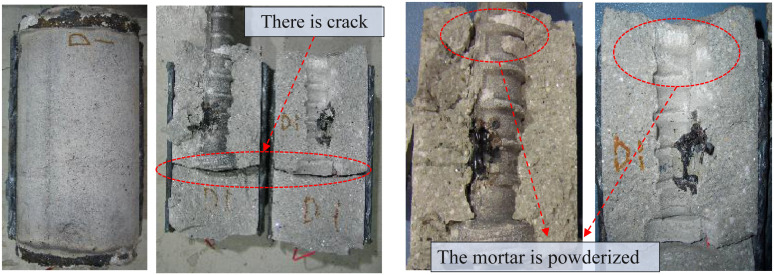
Mortar failure at the stud of specimen D1.

**Fig 29:**
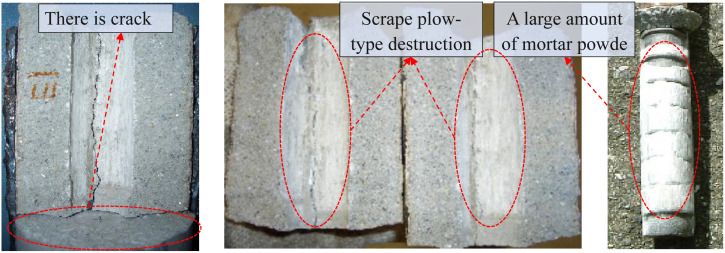
Mortar failure at the stud of specimen E1.

Given the adequate uplift bearing capacity achieved when the end studs of the components are embedded at a depth of 4d, the mortar failure inside the steel tubes in specimens C, D, and E was analyzed, with the results presented in Figs 26–29.

As shown in Fig 26, except for D1, no crack was found between the steel tube and the mortar, while horizontal cracks were detected in all other test pieces. The cracks in specimen D1 did not propagate to the outer layer of the grouting material, since the thicker steel tube effectively constrained the deformation of the grouting material.

The cracks in specimen C4 were located closer to the steel tube backing plate than those in the other specimens, and the position of the horizontal cracks was perfectly coincident with the end of the stud head. In group C, which was fitted with plain round studs, the applied load was primarily borne by the stud heads. The stud heads compressed the surrounding mortar material, eliminating internal voids and resulting in a powdery texture. Under tensile loading, the stud elongated and gradually necked from the outside inward. At a distance exceeding 1d from the stud heads, debonding occurred between the grouting material and the stud, leading to interfacial bond failure, as shown in Fig 27.

In group D, deformed studs with transverse ribs transferred a significant portion of the applied load. Consequently, the grouting material surrounding the studs within the range from the steel tube backing plate to an embedment depth of 2d sustained the most severe pulverization damage, and the grouting material located between the two ribs at a distance of 1d from the steel tube backing plate underwent complete shear failure. The pull-out forces also induced horizontal cracking at the stud base, and internal microcracks were observed in specimen D1 following sectioning, as shown in Fig 28. In specimen E1, the shear failure of the grouting material between the transverse ribs led to the loss of both mechanical interlock and interfacial bonding between the stud and grouting material, which ultimately resulted in the complete pull-out of the stud, as shown in Fig 29.

### 3.7. Comparative analysis of research parameters

**Fig 30:**
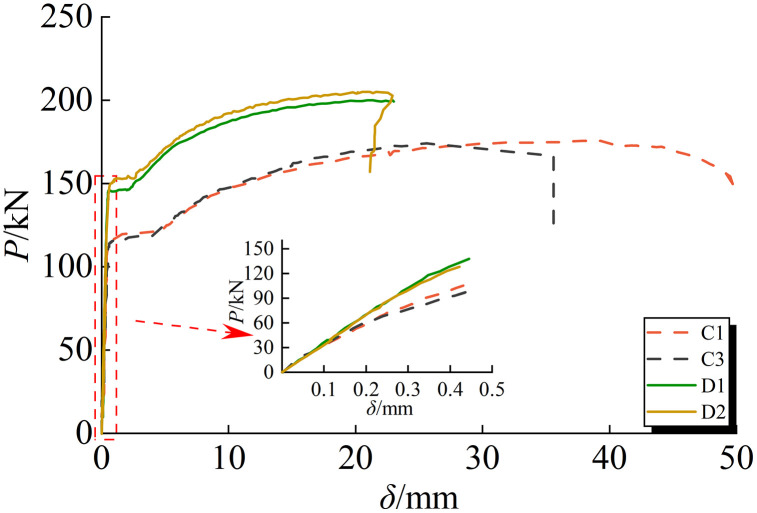
Different steel tube thickness.

**Fig 31:**
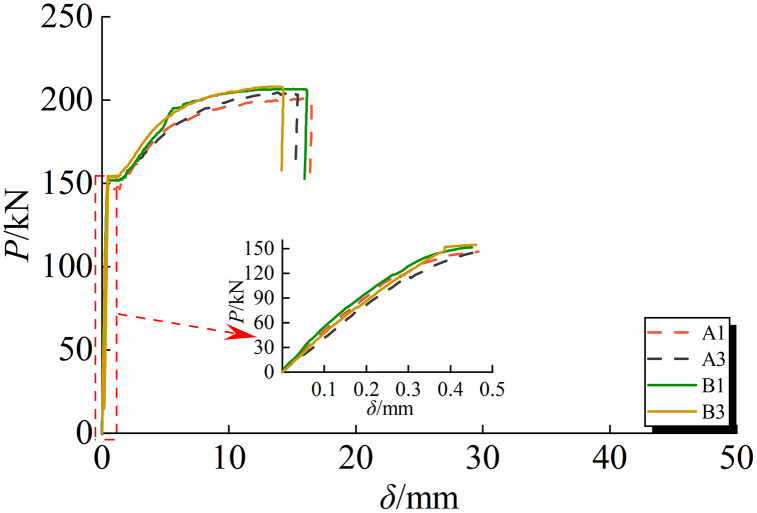
Different head diameter.

**Fig 32:**
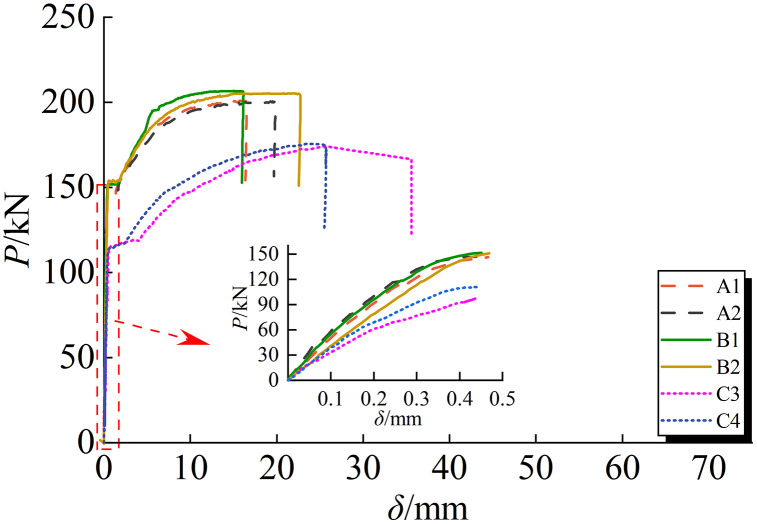
A different diameter of the steel tube backing plate.

**Fig 33:**
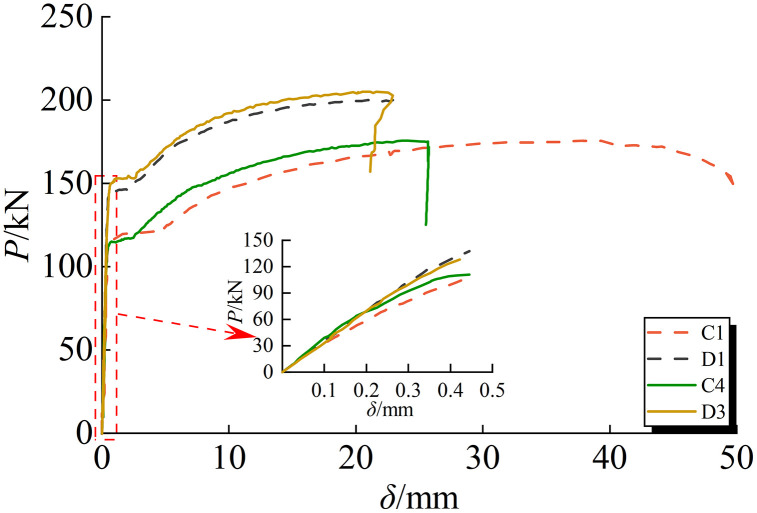
Different steel types of stud.

The control variable method was used to analyze the effects of various parameters on the pullout behaviour of the connection. The results are presented in Figs 30–33, with load on the vertical axis and the average displacement (measured by displacement transducers on both sides of the steel tube) on the horizontal axis. Fig 30 shows the load–deformation curves of specimens with different steel tube wall thicknesses (C1, C3, and D1, D2). Fig 31 presents those for specimens with different stud end diameters (A1, A3, and B1, B3). Fig 32 illustrates those for specimens with different opening sizes of the steel tube base plate (A1, A2; B1, B2; C1, C2). Fig 33 displays those for specimens of different stud steel grades (C1, D1, and C3, D2).

Fig 30 shows that specimens with a steel tube wall thickness of 4.2 mm (C1, D1) exhibit better overall ductility than those with a thickness of 2.8 mm (C3, D2), although their ultimate load capacity is slightly lower. This difference in load capacity is negligible. Fig 31 reveals that the stud head diameter exerts no notable influence on deformation and ultimate load capacity. Fig 32 indicates that the base plate hole diameter has a negligible effect on ultimate load capacity, as seen from the comparisons between Group A1/B1 (28 mm) and A2/B2 (37 mm), and between C4 (37 mm) and C3 (46 mm). Nevertheless, a larger hole diameter leads to greater maximum deformation. Additionally, Fig 32 shows that stud embedment depth has no obvious impact on either ultimate load capacity or deformation (specimens A1, B1, A2, B2). Fig 33 demonstrates that deformed studs effectively restrain overall deformation while maintaining a higher ultimate load capacity.

## 4. Numerical simulation analysis

The STG connector exhibits favourable deformation and failure behaviors under pull-out loading. An FE simulation was performed on STG connectors for structural steel exterior walls using the ABAQUS software. The numerical results were compared with experimental data to validate the feasibility of the proposed FE methodology. These comparisons demonstrated that the FE method can effectively capture the mechanical behaviors of the structural components.

### 4.1. Finite element model

**Fig 34:**
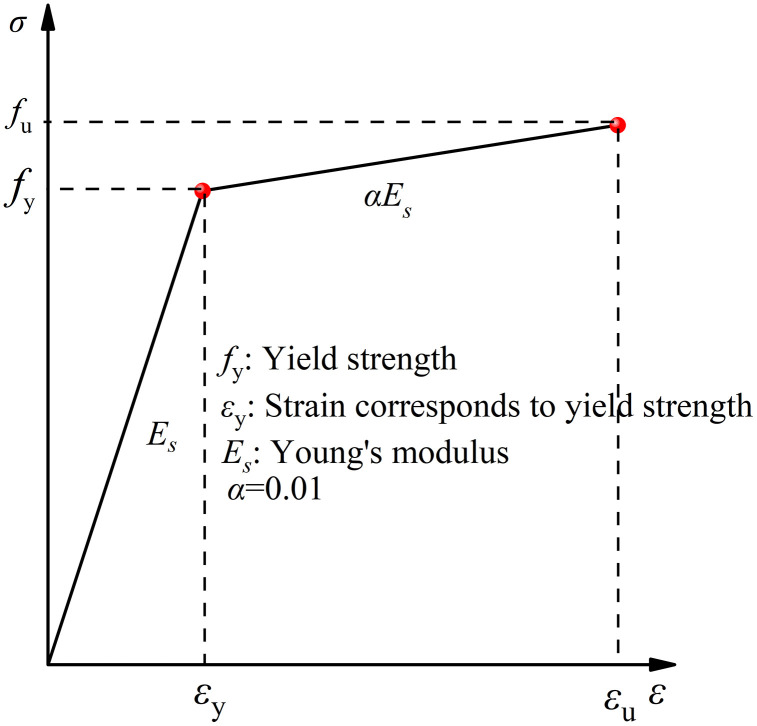
Stress-strain curve of steel.

**Fig 35:**
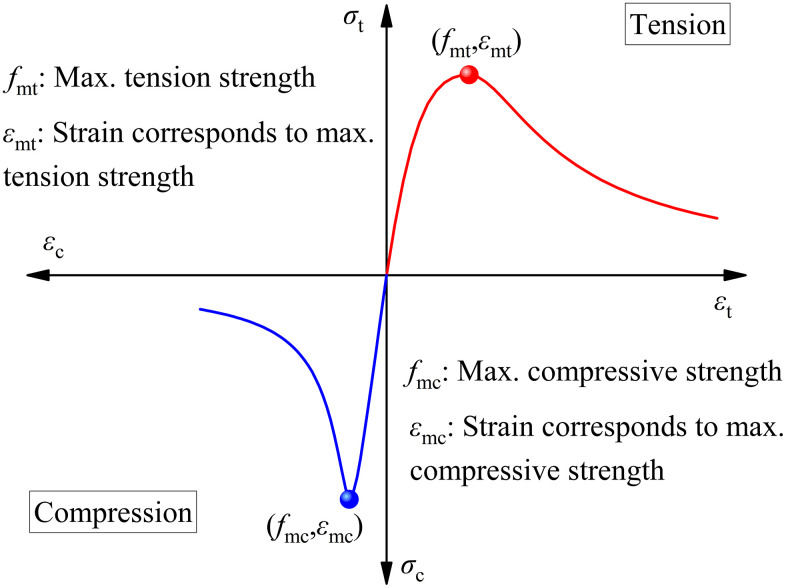
Concrete constitutive model.

The pull-out test results of grout-filled steel tube connections revealed cracking and partial pulverization of the mortar in contact with the studs. This phenomenon can be attributed to the complex interactions between the studs and mortar during the pull-out process, which involves a nonlinear relationship. Given Abaqus’ robust capability in handling such nonlinear contact problems, it was selected for this investigation. Both the studs and steel tubes were modeled as homogeneous elastoplastic materials, adopting a bilinear constitutive model, as shown in Fig 34. The grouting mortar was characterized using the Concrete Damage Plasticity (CDP) model [40–42], which is primarily utilized to simulate material degradation induced by irreversible damage. The constitutive model of the grout material is shown in Fig 35 and Table 6.

**Table 6:** CDP model parameters for grout material.

Dilation Angle *Ψ*/°	Eccentricity *ε*	fb0/fc0 *α*_*f*_	K	Viscosity Parameter *µ*
30	0.1	1.16	2/3	0.0005

**Fig 36:**
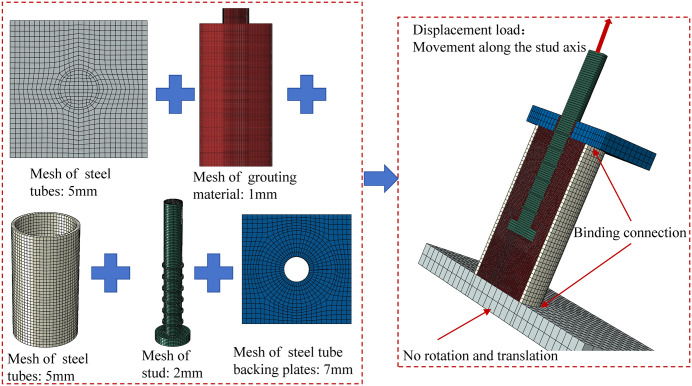
Finite element modeling.

The FE model is shown in Fig 36. In the FE simulation of the STG connectors, the interaction mechanisms (e.g., between the grouting material and studs) were critical. To simulate these interactions, transverse ribs were modeled on the contact surface between the mortar and studs. Element sizes exerted a significant influence on the results. For element selection, the three-dimensional solid element C3D8R (an eight-node linear hexahedral reduced-integration element) was adopted for the studs, grouting material, steel tubes, and steel plates to ensure accuracy. For mesh generation, multiple simulation trials and mesh size adjustments were performed. In particular, mesh densification was required for the grouting material and studs. The final mesh sizes were set as follows: 1 mm for the grouting material, 2 mm for the studs, 5 mm for the steel tubes, 7 mm for the steel tube backing plates, and 10 mm for the bottom plates, as shown in the left panel of Fig 36. For the interactions, contact definitions were applied to both the steel tube-grouting material and stud-grouting material interfaces. Hard contact was assigned for the normal direction, while Coulomb friction with a friction coefficient of 0.57 was specified for the tangential direction [43]. Tie constraints were imposed at the welded interfaces, namely between the bottom plate and steel tube, and between the steel tube backing plate and steel tube. For boundary conditions, the lower end of the component’s bottom plate was fixed (via a rigid connection), and displacement-controlled loading was applied to the upper end of the stud, with the magnitude consistent with the experimental conditions. The analysis model of the STG connector is shown in the right panel of Fig 36.

### 4.2. Comparison of numerical analysis results

#### 4.2.1. Comparison of load-deformation curves.

**Fig 37:**
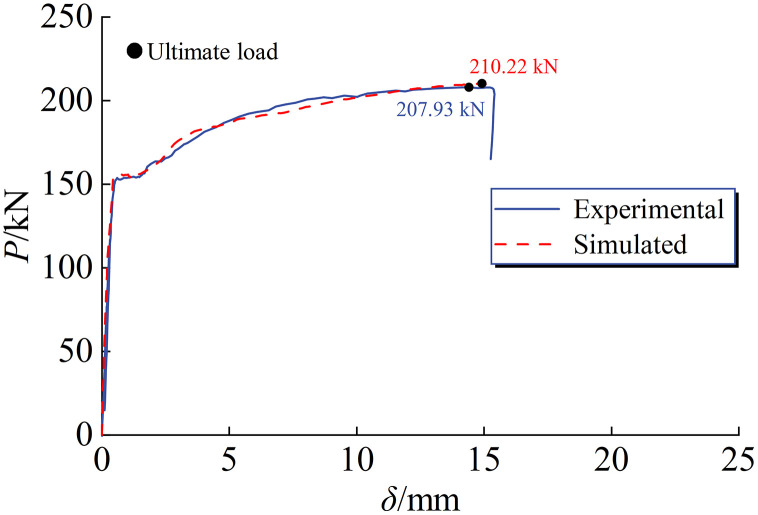
Comparison of load-displacement curves of specimen A5.

**Fig 38:**
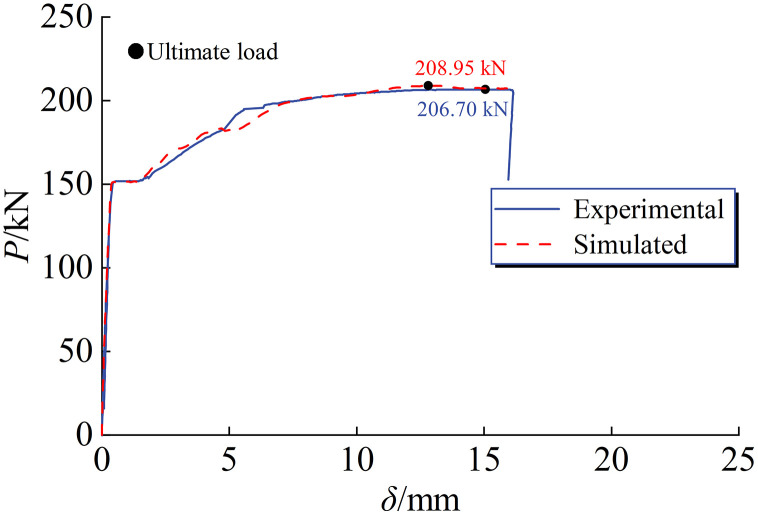
Comparison of load-displacement curves of specimen B1.

**Fig 39:**
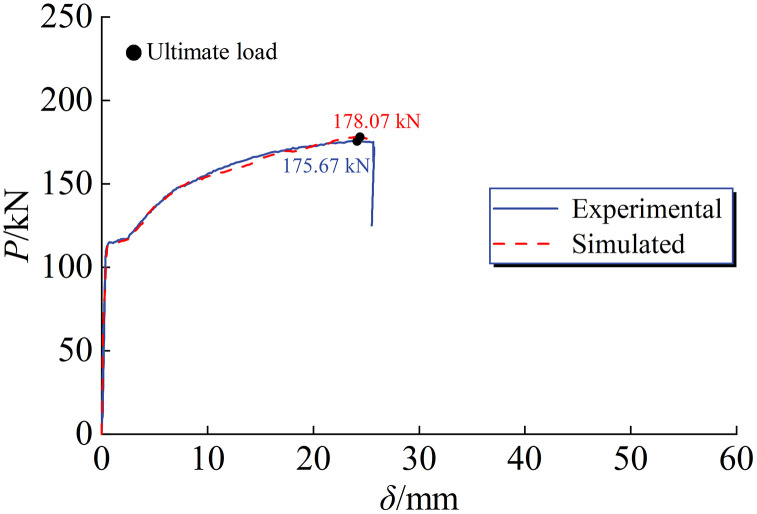
Comparison of load-displacement curves of specimen C4.

**Fig 40:**
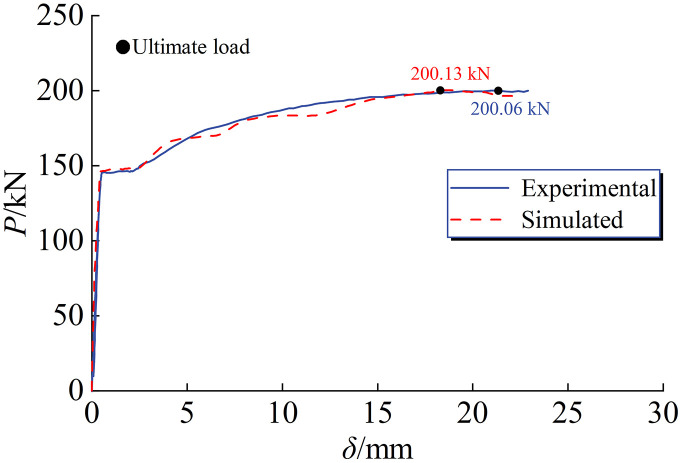
Comparison of load-displacement curves of specimen D1.

**Fig 41:**
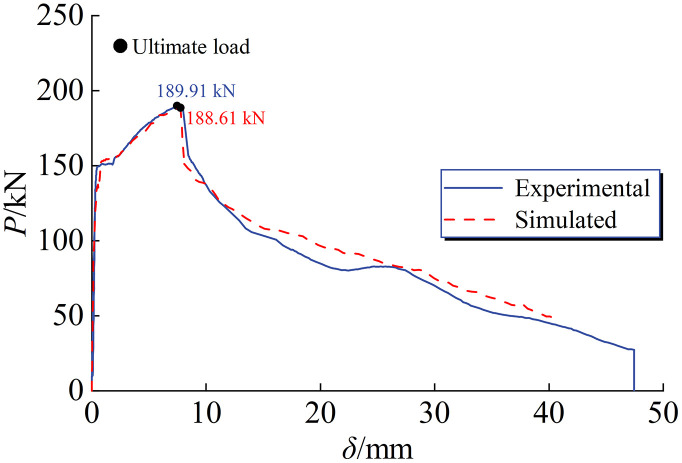
Comparison of load-displacement curves of specimen E1.

The axial deformation and applied forces were extracted for selected STG connectors (specimens A5, B1, C4, D1, and E1), with extraction points matching those in the test. Simulation results were compared with experimental findings, as shown in Figs 37–41.

From Figs 37–41, the numerical simulation results are compared with the experimental test data. The simulated load-displacement curves exhibit a close match with the experimental counterparts, with consistent response trends observed throughout the elastic, yield, and hardening stages.

#### 4.2.2. Comparison of mortar failure inside specimens.

**Fig 42:**
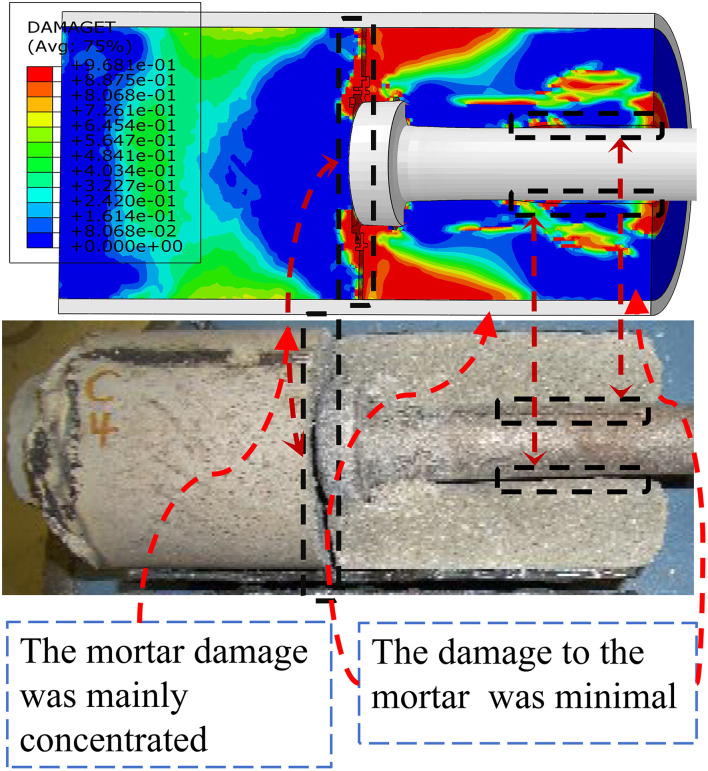
Comparison of mortar failure in specimen C4.

**Fig 43:**
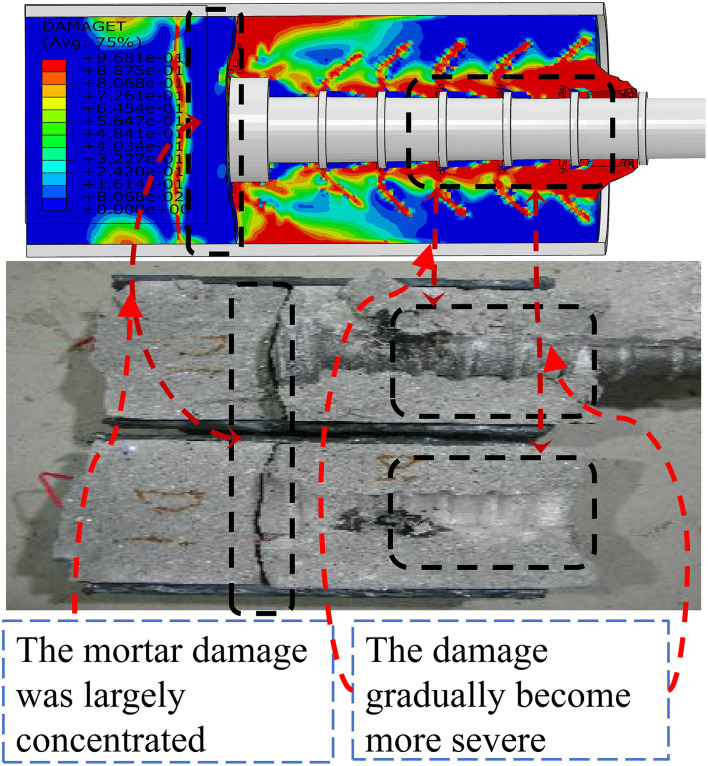
Comparison of mortar failure in specimen D1.

**Fig 44:**
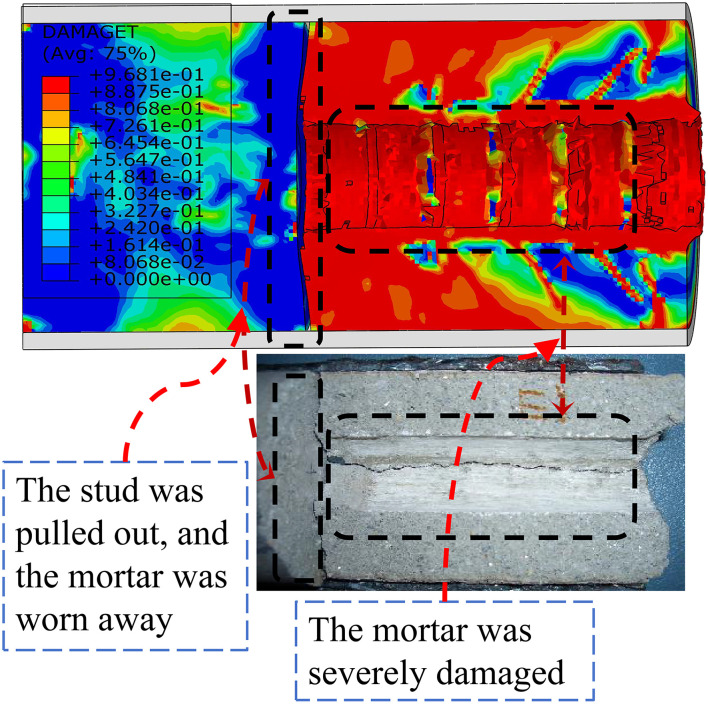
Comparison of mortar failure in specimen E1.

Figs 42–44 shows mortar failure in selected specimens (C4, D1, and E1). The FE analysis revealed the presence of nearly horizontal cracks in the grouting material at the stud head, consistent with experimental observations.

For the smooth steel stud specimen C4, the interaction between the stud and grouting material (mortar) was minimal. Simultaneously, tensile loading induced a reduction in the stud diameter, creating gaps between the stud and the surrounding mortar. Consequently, mortar damage around the stud was negligible, with damage predominantly concentrated at the stud head, as shown in Fig 42.

For the headed deformed stud specimen D1, mortar damage was concentrated both at the stud head and along the shaft, intensifying progressively from the head outward. This phenomenon aligned well with the experimental results, indicating that within the embedment depth of 2d, the degree of mortar damage remained consistent, as shown in Fig 43. For the headless deformed stud specimen E1, the stud was fully pulled out, exhibiting severe mortar damage along the shaft and a plough-like failure mode, as shown in Fig 44.

#### 4.2.3. Comparison of steel tube strains.

**Fig 45:**
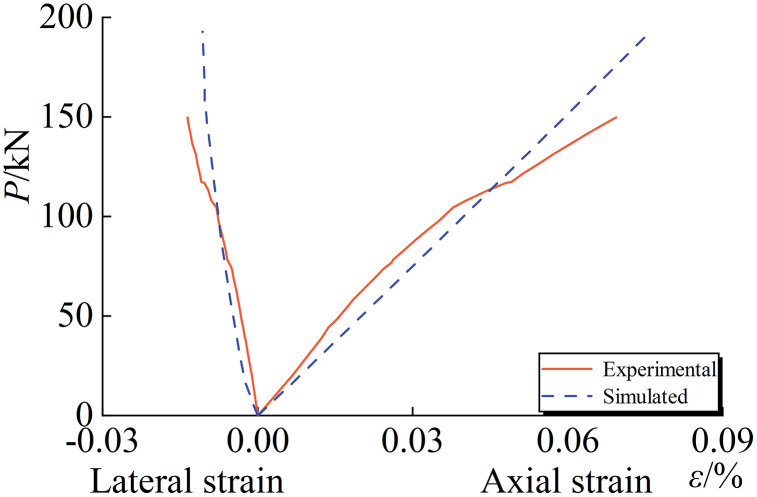
Comparison of strain curves for specimen A5.

**Fig 46:**
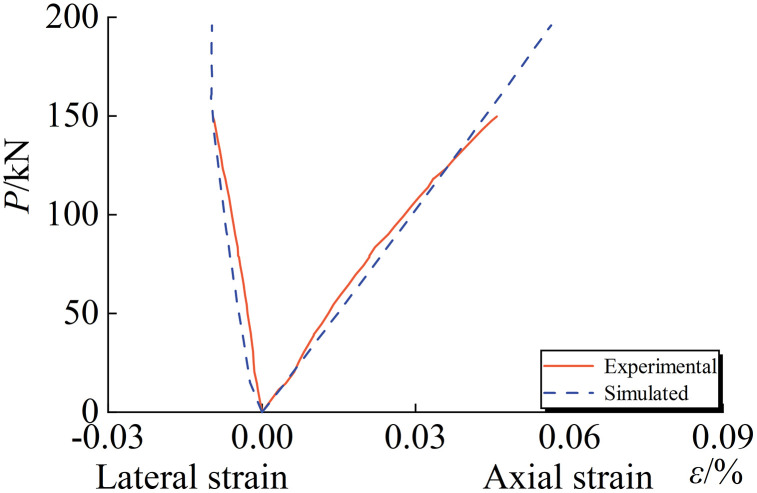
Comparison of strain curves for specimen B1(4d).

**Fig 47:**
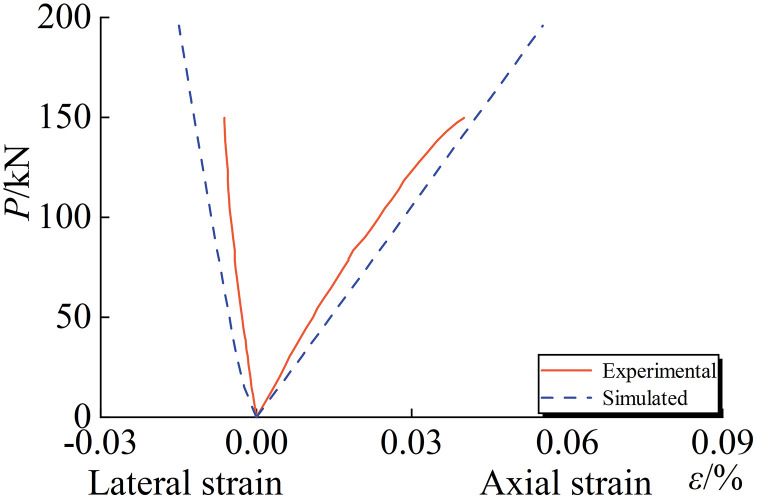
Comparison of strain curves for specimen B1(8d).

**Fig 48:**
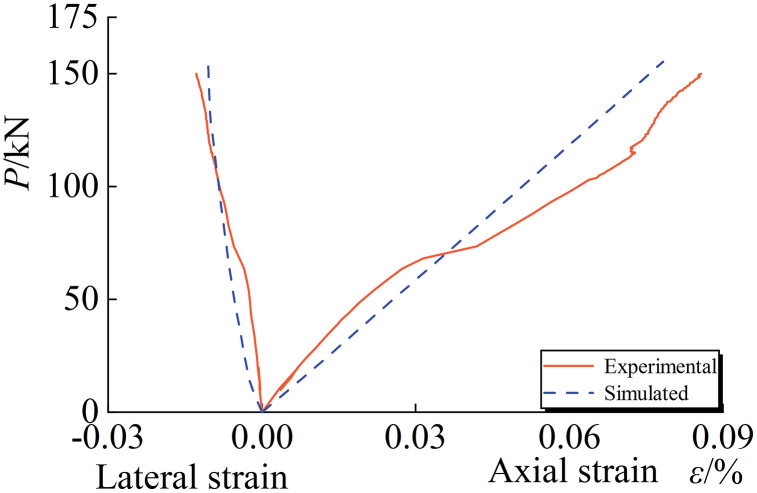
Comparison of strain curves for specimen C4.

**Fig 49:**
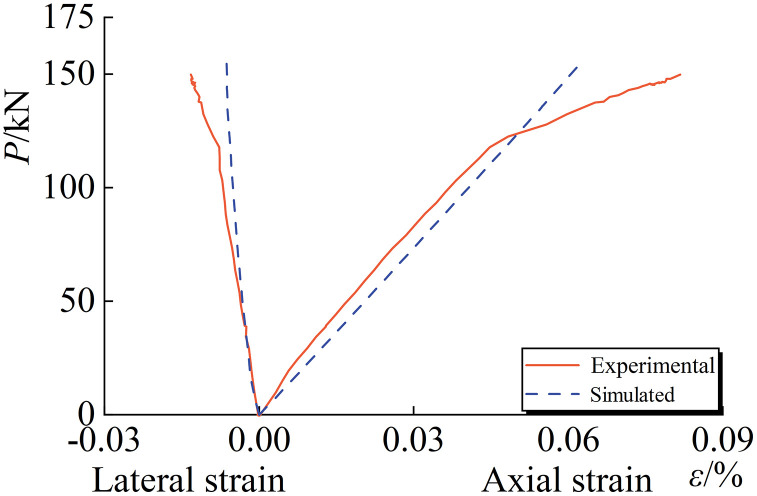
Comparison of strain curves for specimen D1.

**Fig 50:**
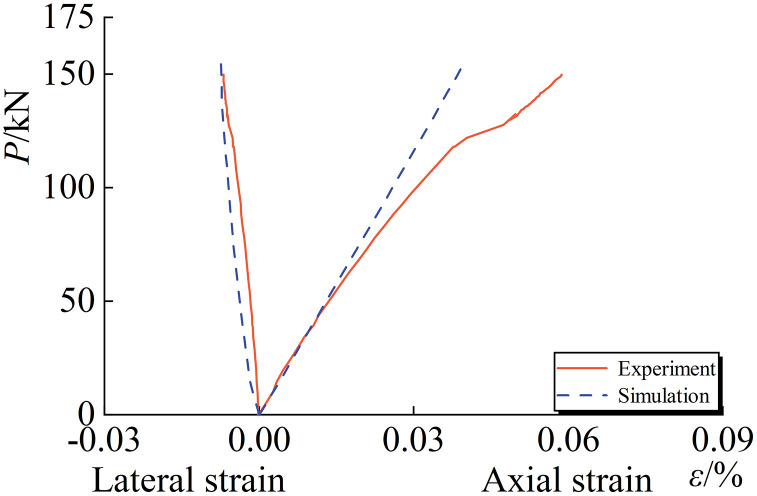
Comparison of strain curves for specimen E1.

The FE analysis and experimental results comparing the load-strain curve relationship at the outermost layer of some test specimens’ steel tubes are shown in Figs 45–50.

As shown in the comparison of steel tube strains, the FE simulation results are in good agreement with the experimental strain data measured on the outer surface of the steel tube. The initial strain growth rates derived from both approaches are nearly identical, indicating that the proposed FE model can accurately reproduce the initial stiffness of the component. However, when the applied load exceeds 120 kN, a notable discrepancy arises between the simulated and experimental axial strain curves for specimens A5, C4, D1, and E1. This discrepancy primarily stems from the inability of the FE model to fully capture the strain concentration occurring on the steel tube surface, which is induced by localised buckling or manufacturing defects during the experimental tests. Additionally, the reduced sensitivity of strain gauges in the large-deformation stage may have contributed to such discrepancies. Meanwhile, Figs 45–50 indicate minimal variation in the slope of the FE simulation curves, attributable to the steel tube remaining within the elastic range throughout the entire deformation process.

## 5. Theoretical analysis

### 5.1. Stress state of grouting material

**Fig 51:**
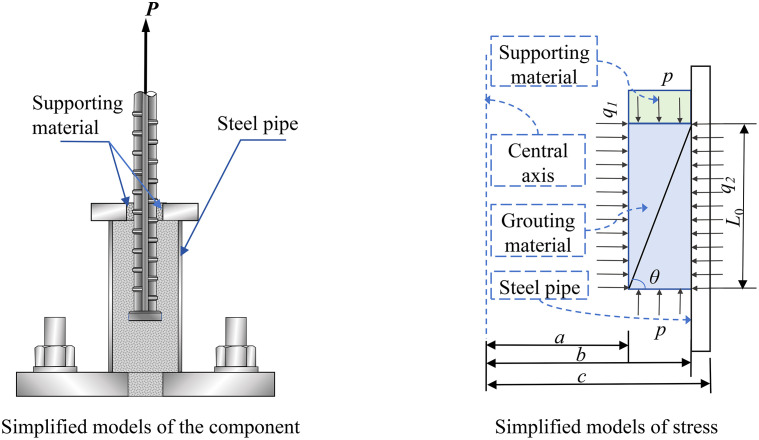
Stress state of mortar near steel tube backing plate.


To evaluate the yield load of the studs, the grouting material in the regions adjacent to the stud head and steel tube backing plate was modeled. The stress distribution from the steel tube to the adjacent mortar is illustrated in Fig 51. A cylindrical coordinate system *(r,* θ, *z)* was employed, with the z-axis aligned along the central axis of the specimen. Parameters *a*, *b*, and *c* denote the radial distances from the central axis to three characteristic positions, respectively: *a*: the inner wall of the steel tube backing plate hole, *b*: the inner wall of the steel tube, and *c*: the outer wall of the steel tube. It is assumed that the vertical load *P* is fully transmitted through the supporting materials, namely the stud head or the steel tube backing plate. Since friction exerted no contribution to load transmission, it was neglected in the model. In the case of multiple supporting materials, the quantity of such materials is determined based on their respective areas to ensure uniform distribution of the vertical load. The vertical stress, p, can be calculated using Eq. (1), where *P* is the vertical load in the pull-out test, *N* is the number of supporting materials, $A_{r}$ is the supporting area of the supporting material, $A_{r}=\pi (b^{\text{2}}-a^{\text{2}})$.

$$\begin{array}{c} p=\frac{P}{N\cdot A_{r}} \end{array}$$ (1)

Based on previous elastic theory studies [44,45], the radial stress ($\sigma_{r}$) and lateral stress ($\sigma_{\theta }$) in the mortar adjacent to the supports were modeled as stresses induced by internal pressure ${\text{q}}_{\text{1}}$, as shown in the left panel of Fig 51. The internal pressure is calculated by Eq. (2), where *p* denotes the vertical stress, and α denotes the lateral pressure coefficient.


$$\begin{array}{c} q_{\text{1}}=\alpha \cdot p \end{array}$$ (2)

The restraining effect (i.e., the Bauschinger effect) on the components is considered in this study, with the specific implementation procedure described as follows. The confining effect of the steel tube is modeled as an external pressure. Taking the compressive stress in the mortar as positive at *r* = *b*, the corresponding stress distribution within the mortar can be derived from elastic theory and is expressed as follows:


$$\begin{array}{c} \sigma_{z}=p \end{array}$$

$$\begin{array}{c} \sigma_{r}=q_{\text{2}} \end{array}$$

$$\begin{array}{c} \sigma_{\theta }=\frac{-\text{1}}{b^{\text{2}}-a^{\text{2}}}[\text{2}a^{\text{2}}\cdot q_{\text{1}}-(a^{\text{2}}+b^{\text{2}})q_{\text{2}}] \end{array}$$ (3)

The crushing zone of the grouting material is defined in terms of the width of the crushing region and the height *L*_0_, where *L*_0_ is determined by a straight line at an angle *θ*. The angle *θ* corresponds to the shear failure angle described by the Mohr-Coulomb strength criterion and is correlated with the internal friction angle *φ* of the mortar via the relation 2θ=π/2+φ. Focusing on the extracted *L*_0_ zone and neglecting the influence of surrounding regions, the relationship between the vertical stress p and the confining stress exerted by the steel tube is established by equating the radial deformation of the mortar ($u_{c}$) to that of the steel tube ($u_{s}$). Based on Eqs. (2) and (3), the radial deformation of the mortar at r = b can be expressed as follows:


$$\begin{array}{c} u_{c}=(b-a)\cdot \varepsilon_{\theta }=\frac{\left(b-a\right)}{E_{c}}[\sigma_{\theta }-v_{c}(\sigma_{z}+\sigma_{r})] \end{array}$$

$$\begin{array}{c} =\frac{\left(b-a\right)}{E_{c}}\left\{\frac{-\text{1}}{b^{\text{2}}-a^{\text{2}}}[\text{2}a^{\text{2}}\cdot q_{\text{1}}-(a^{\text{2}}+b^{\text{2}})q_{\text{2}}]-v_{c}(p+q_{\text{2}})\right\} \end{array}$$

$$\begin{array}{c} =\frac{\left(b-a\right)}{E_{c}}\left\{\frac{-\text{1}}{b^{\text{2}}-a^{\text{2}}}[\text{2}a^{\text{2}}\cdot \alpha \cdot \text{p}-(a^{\text{2}}+b^{\text{2}})q_{\text{2}}]-v_{c}(p+q_{\text{2}})\right\} \end{array}$$

$$\begin{array}{c} =\frac{\left(b-a\right)}{E_{c}}\left\{-\left[\frac{\text{2}a^{\text{2}}}{b^{\text{2}}-a^{\text{2}}}\cdot \alpha +v_{c}\right]\cdot p+\left[\frac{b^{\text{2}}+a^{\text{2}}}{b^{\text{2}}-a^{\text{2}}}-v_{c}\right]\cdot q_{\text{2}}\right\} \end{array}$$ (4)

Where $E_{c}$ denotes the elastic modulus of the mortar, and $v_{c}$ denotes its Poisson’s ratio. In addition, the radial deformation $u_{s}$ of the steel tube (considering only the lateral direction) under internal pressure $q_{\text{2}}$ is calculated using the following formula:


$$\begin{array}{c} \sigma_{\theta }=-\frac{b-a}{c-b}\cdot q_{\text{2}} \end{array}$$ (5)

$$\begin{array}{c} \;u_{s}=\frac{b-a}{E_{s}}\cdot \frac{b-a}{c-b}\cdot q_{\text{2}} \end{array}$$ (6)

By satisfying the deformation condition $u_{c}=u_{s}$ and combining Eqs. (4) and (6), the following equation can be obtained:


$$\begin{array}{c} \frac{b-a}{E_{s}}\cdot \frac{b-a}{c-b}\cdot q_{\text{2}}=\frac{\left(b-a\right)}{E_{c}}\left\{-\left[\frac{\text{2}a^{\text{2}}}{b^{\text{2}}-a^{\text{2}}}\cdot \alpha +v_{c}\right]\cdot p+\left[\frac{b^{\text{2}}+a^{\text{2}}}{b^{\text{2}}-a^{\text{2}}}-v_{c}\right]\cdot q_{\text{2}}\right\} \end{array}$$

$$\begin{array}{c} \left[\frac{b^{\text{2}}+a^{\text{2}}}{b^{\text{2}}-a^{\text{2}}}-v_{c}+\frac{E_{c}}{E_{s}}\cdot \frac{b-a}{c-b}\right]\cdot q_{\text{2}}=\left[\frac{\text{2}a^{\text{2}}}{b^{\text{2}}-a^{\text{2}}}\cdot \alpha +v_{c}\right]\cdot p \end{array}$$

$$\begin{array}{c} q_{\text{2}}=\frac{\frac{\text{2}a^{\text{2}}}{b^{\text{2}}-a^{\text{2}}}\cdot \alpha +v_{c}}{\frac{b^{\text{2}}+a^{\text{2}}}{b^{\text{2}}-a^{\text{2}}}-v_{c}+\frac{E_{c}}{E_{s}}\cdot \frac{b-a}{c-b}}\cdot p \end{array}$$ (7)

Where $A=\frac{\text{2}a^{\text{2}}}{b^{\text{2}}-a^{\text{2}}}\cdot \alpha +v_{c},\;\text{B}=\frac{b^{\text{2}}+a^{\text{2}}}{b^{\text{2}}-a^{\text{2}}}-v_{c},\;\text{C}=\frac{E_{c}}{E_{s}}\cdot \frac{b-a}{c-b}.$

Therefore, Eq. (7) can be simplified as:


$$\begin{array}{c} q_{\text{2}}=\frac{A}{B+C}\cdot p \end{array}$$ (8)

**Fig 52:**
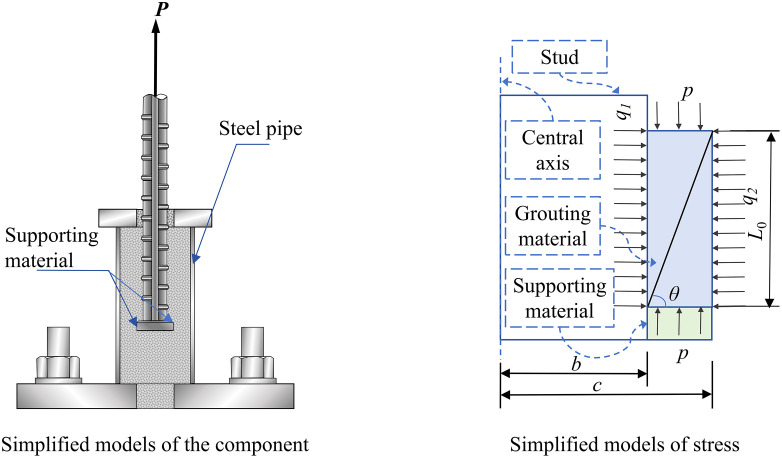
Mortar stress state near the end of the stud.

It is assumed that the stress distribution of the mortar near the stud heads follows the pattern shown in Fig 52. Treating the transverse ribs as part of the supporting components for the heads of the deformed studs, the equivalent number of supporting components for the stud heads can be approximated as the ratio of the sum of the bearing area of the transverse ribs and the bearing area of the stud heads to the bearing area of the stud heads, as summarized in Table 7.

**Table 7:** Approximate number of supporting materials for end-deformed studs.

End diameter/mm	Stud diameter/mm	End support area/mm^2^	Transverse rib number	Supporting area of a single transverse rib/mm^2^	Number of supporting materials
35 (Group A/D)	22	582	6	105	2
35(Group B)	22	582	12	105	3
44(Group A)	22	1140	6	105	1.5
44(Group B)	22	1140	12	105	2

In the stress state diagram shown in Fig 52, the distance from the *z*-axis to the stud surface is denoted as *b*, while the distance from the *z*-axis to the outer edge of the supporting material is denoted as *c*. Based on the relationship between the applied vertical stress *p* and the confining stress $q_{\text{2}}$ acting on the stud, the following equation can be derived:


$$\begin{array}{c} q_{\text{2}}=\frac{A}{B+C}\cdot p \end{array}$$ (9)

Where $A=\frac{c^{\text{2}}+b^{\text{2}}}{c^{\text{2}}-b^{\text{2}}}\cdot \alpha -v_{c},\;B=\frac{{\text{2}b}^{\text{2}}}{c^{\text{2}}-b^{\text{2}}}+v_{c},\;C=\frac{E_{c}}{E_{s}}\cdot \frac{b}{c-b}.$

### 5.2. Application of Mohr-Coulomb failure criterion

For the stress state of the mortar adjacent to the supporting materials obtained in the preceding section, the Mohr-Coulomb failure condition is applied as follows. In Eq. (10), $\tau_{n}$ denotes the shear stress at mortar fracture, $\tau_{\text{0}}$ denotes the adhesion stress of the mortar, $\sigma_{n}$ denotes the vertical stress at mortar fracture, and φ denotes the internal friction angle of the mortar.


$$\begin{array}{c} \tau_{n}=\tau_{\text{0}}+\sigma_{n}\text{tan}\varphi \end{array}$$ (10)

The acting stresses p and $q_{\text{2}}$ at the onset of mortar failure, together with the compressive strength $\sigma_{B,c}$ of mortar, are adopted as the failure criteria, from which the following can be derived:


$$\begin{array}{c} p=\frac{\text{1}+sin\varphi }{\text{1}-sin\varphi }q_{\text{2}}\sigma_{B,c} \end{array}$$ (11)

Eq. (12) is obtained via simplification, from which the vertical load can be calculated.


$$\begin{array}{c} P_{a}=\frac{N\cdot A_{r}\cdot \sigma_{B,c}}{\text{1}-\frac{\text{1}+\text{sin}\varphi }{\text{1}-\text{sin}\varphi }\cdot \frac{A}{B+C}} \end{array}$$ (12)

### 5.3. Comparison between test results and theoretical values

**Fig 53:**
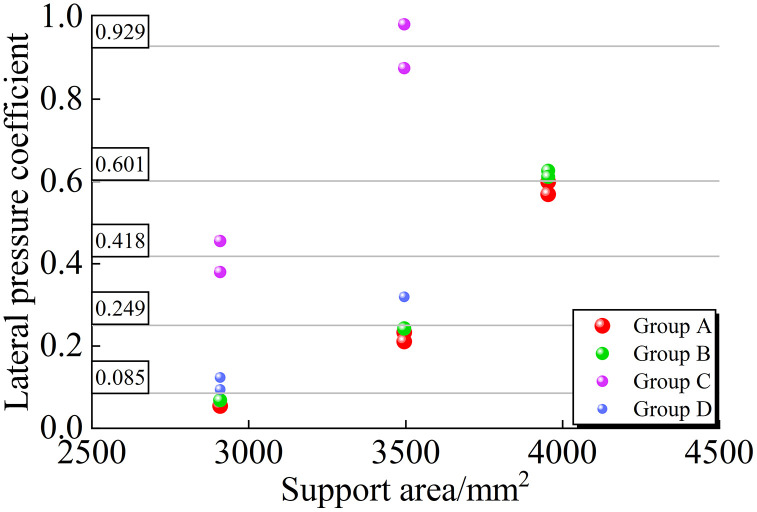
Distribution of lateral pressure coefficient values near the steel tube backing plate.

**Fig 54:**
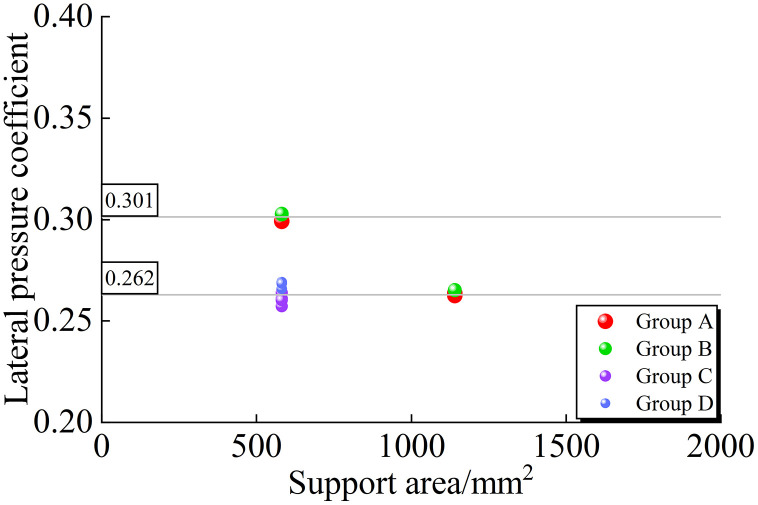
Distribution of lateral pressure coefficient values near the stud head.

Parameter settings: Based on previous studies [46,47], the internal friction angle of the mortar ranges from 30° to 50°. For calculation convenience, this parameter is assumed to be 45° herein. The Poisson’s ratio of the mortar is considered to be 0.2, and the compressive strength is defined as the average of the experimental results. The elastic modulus *E* of steel is set to 2.05 × 10⁵ N/mm², while the elastic modulus of the mortar is also taken as the average of the test data. The Poisson’s ratio of steel is assumed to be 0.3. The lateral pressure coefficient *α* in Eq. (2) was calculated using Eq. (12), based on the yield load of the specimens obtained from the experimental tests. In this study, the lateral pressure coefficient was determined by analyzing the mortar adjacent to the steel tube backing plate and that adjacent to the stud head, as shown in Figs 53 and 54.

**Fig 55:**
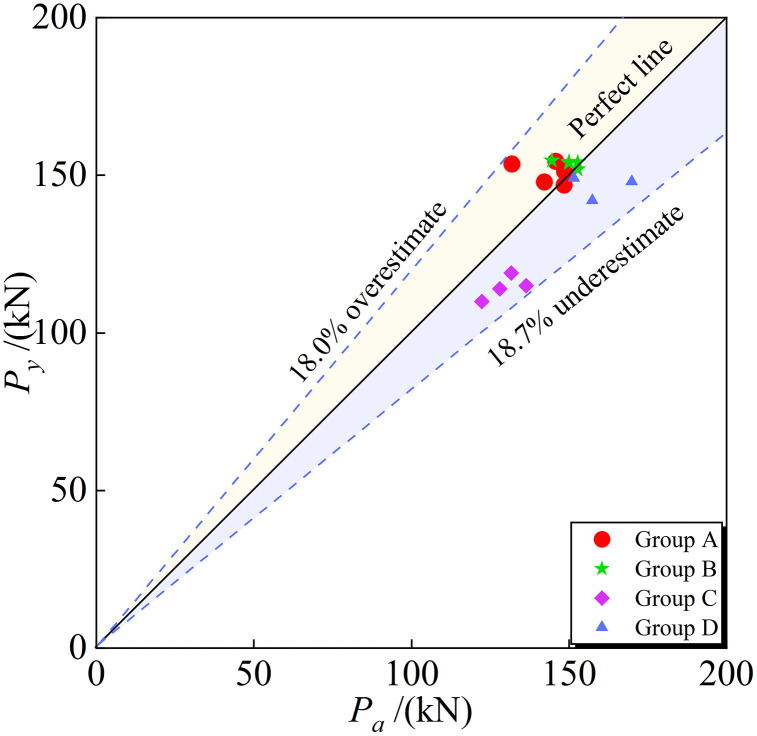
The correlation between Py and Pa near the steel tube packing plate.

**Fig 56:**
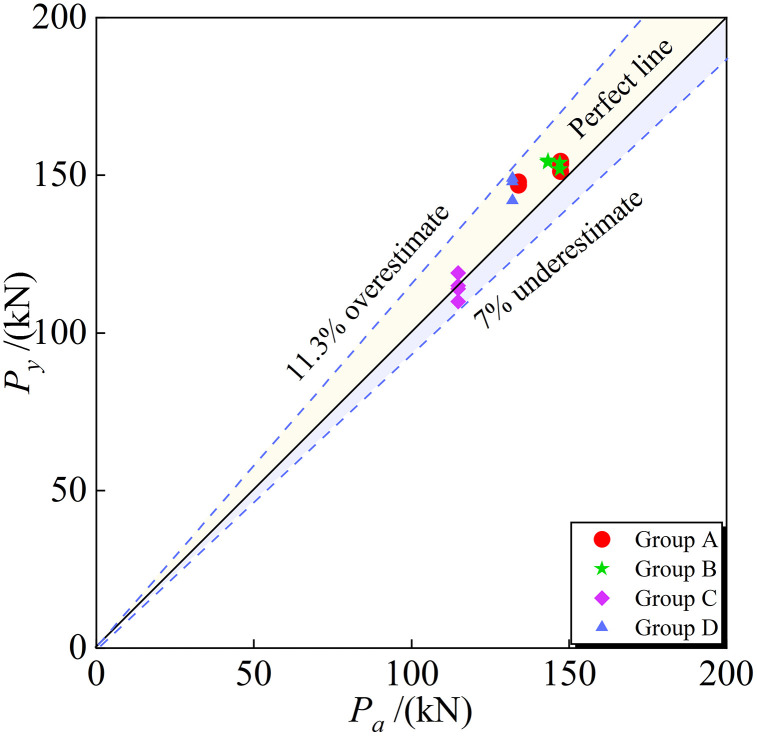
The correlation between Py and Pa near the headed stud.

Yield load evaluation: The yield load of the test specimens is evaluated based on the acting stress in the mortar, with yield loads calculated separately for the steel tube side and the stud end. The results indicate that the yield load is governed by the studs, which is consistent with both the observed failure modes and the experimental analysis. Figs 55 and 56 show the correlation between the yield load *P*_*y*_ experimentally measured and the yield load *P*_*a*_ calculated from Eq. (12). A comparison of the calculated and experimental values for the mortar adjacent to the steel tube bearing plate reveals a maximum error of 18.7%. This error is primarily attributed to the relatively low strength of the smooth round steel in the specimens of group C, where the interaction between grouting material and stud is relatively minor. The comparison between the calculated and experimental values for the mortar near the stud head exhibits a maximum error of 11.3%.

## 6. Summary and conclusions

To improve the connection technology between the exterior walls and the main structure, this study proposes a novel small-diameter steel tube-stud grouting connector designed for such joints. The tensile performance of the proposed connector was investigated via a combination of pull-out tests, numerical simulations, and theoretical analysis. The research first examined the effects of key parameters, including steel type of stud, stud embedment depth, and failure modes. On this basis, numerical simulations were employed to analyse the stress distribution within the connector and validate the results against experimental data. Finally, a theoretical method for calculating the yield bearing capacity of the proposed grouting connector under tensile loading was established. The main conclusions of this study are summarized as follows:

(1) Pull-out tests revealed that all specimens (excluding E1) successfully resisted stud pull-out failure. It demonstrated that the STG connectors maintain reliable mechanical performance even when partial mortar damage occurs. As the hole diameter of the steel tube backing plate increases and the tube thickness decreases, the specimen stiffness tends to decline, with no significant differences in mechanical performance observed. Additionally, it was found that round steel studs exhibit an end effect. Therefore, the solution presented in this study, which features shallow stud embedment and low-strength grout, can effectively cut material expenditure, streamline construction work, raise field assembly efficiency, and comply with the development needs of industrialized building systems.
(2) In the numerical simulations, the stiffness and load-bearing capacity reflected in the load-deformation curves were in close agreement with the experimental results, and the predicted failure modes were highly consistent between the tests and the FE analysis. This confirms that the concrete damaged plasticity model is suitable as the constitutive model for the grouting material. The findings provide valuable references for the finite element analysis of grouted connections.
(3) The yield bearing capacity of the pull-out specimens was evaluated using a formula derived from the Mohr–Coulomb strength theory and elastic theory. The predicted yield load showed good agreement with the experimental results. Compared with predictions based on the stress distribution of mortar around the steel tube base plate, evaluating the yield load using the stress state at the stud end yields lower errors, with a maximum error of 11.3%. This theoretical method fills research gaps in the theoretical analysis of steel structural grouted connections and refines the mechanical calculation framework for such connection systems.
(4) This study demonstrates that low- to medium-strength grout can deliver reliable pullout performance, challenging the conventional design approach of adopting high-strength grout and large embedment depth. It clarifies the synergistic load-transfer mechanism consisting of steel tube confinement, stud end bearing, and localized grout support, and provides key experimental data and theoretical support for subsequent parametric optimization, numerical modelling, and further research on such connections.

## Supporting information

S1 File Data. (ZIP)
