# Supplementary material for: Research on the anti-pull performance of a novel small-diameter steel tube-stud grouting connector
Source: PLoS One. 2026 Jul 22;21(7):e0353244. doi: 10.1371/journal.pone.0353244 (PMC13390846; doi:10.1371/journal.pone.0353244)
Supplement: Attachment — Submitted filename: Response to Reviewers.docx [file pone.0353244.s003.docx]

Dear Editors and Reviewers:

Thank you for your letter and for the reviewers’ comments concerning our manuscript entitled “Research on the anti-pull performance of a novel small-diameter steel tube-stud grouting connector”. Those comments are all valuable and very helpful for revising and improving our paper, as well as providing important guiding significance to our research. We have studied comments carefully and have made corrections which we hope will meet with approval. The main corrections in the paper and the responds to the reviewer’s comments are as follows: Responds to the reviewer’s comments:

**Reviewer #1: General Comments:
This manuscript investigates the pull-out performance of steel tube–stud grouting connectors, with research parameters including embedment depth, stud steel type, steel tube thickness, stud end diameter, and the diameter of the backing plate hole. Based on the Mohr–Coulomb strength criterion and elastic theory, an analytical expression for the yield bearing capacity of connectors under tensile pull-out is developed by modeling the stress state of the surrounding mortar. However, the manuscript is poorly organized, and numerous issues need to be addressed before it can be considered for publication.**

**1. The key words fail to present the main content of this article. It is suggested to replace the first key word.**

**Response:** Thank the expert for the comments. We replaced the first keyword with a more appropriate one and added a keyword. The revised keywords accurately reflect the main content of the revised article, as shown below. The revised keywords: Steel tube-stud grouting connector between steel frame and external wall; Pull-out performance; Finite element analysis; Mohr-Coulomb strength theory; Yield bearing capacity

**2. There is no head diameter in Figure 4. The expression in Figure 4 should correspond to that in Table 1.**

**Response:** Thank the expert for the comments. We have supplemented the parameter of head diameter in Figure 4 and unified the symbol and expression format of Figure 4 to be consistent with Table 1 in the revised manuscript.

**Fig. 4.** Geometric structure diagram of the specimen

**3. Have duplicate specimens been set up, with only one specimen of each type. How can accidental errors be avoided? (Section 2.1)**

**Response:** Thank the expert for the comments. In this study, a total of 18 specimens were designed and divided into 5 groups (A, B, C, D, E) for parametric comparison. Multiple parallel specimens were arranged within each group. Key parameters (stud embedment depth, steel tube wall thickness, base plate hole diameter in the steel tube, stud type, and stud end diameter) each included 2 to 5 parallel specimens, rather than a single-specimen design. All grouting materials and steel were from the same batch. The specimens and test blocks were cured under the same conditions and loaded on the same day to minimize material variability. All specimens were subjected to the same loading rate, preloading cycles, and loading until failure, thereby eliminating accidental errors caused by different loading procedures.

**4. The diameter (d) of the stud should be supplemented in Table 1.**

**Response:** Thank the expert for the comments. We have supplemented the diameter d of the stud in Table 1 in the revised manuscript.

**5. The diameter of the steel pipe should be indicated in Table 1.**

**Response:** Thank the expert for the comments. We have indicated and supplemented the diameter of the steel tube in Table 1 in the revised manuscript.

**6. Pictures of the tensile tests of stud bolts and steel pipes should be supplemented in Section 2.2.**

**Response:** Thank the expert for the comments. Due to an oversight during the experimental recording process, high-definition physical photographs of the tensile tests on the studs and steel tubes were not retained at the earlier stage. However, the relevant mechanical property data (yield strength, tensile strength, elastic modulus, elongation at fracture, etc.) have been tested in strict accordance with the standard (GB/T 228.1-2021) and are fully presented in Tables 2 and 3. The data are authentic and reliable. To improve the content of the paper, we have supplemented schematic diagrams of the tensile tests on the studs and steel tubes, along with dimensional annotations of the specimen geometry, in Section 2.2. Detailed written descriptions of the test procedure, loading method, and data acquisition method have also been provided, clearly presenting the entire process of material mechanical property testing and meeting the traceability requirements of the experiments. In future studies, we will strictly standardize the recording of experimental images to avoid similar oversights. Once again, we thank the reviewers for their guidance. The above revisions have been made in the revised manuscript.

|  | |  |
| --- | --- | --- |
| (a) Schematic diagram of the specimen | |  |
|  | |  |
| (b) Loading diagram of the stud test | |  |
| **Fig. 5.** Stud tensile test | |  |
|  |  | |
| (a) Schematic diagram of the specimen | (b) Loading diagram of the steel tube test | |
| **Fig. 6.** Steel tube tensile test | | |

**7. According to the provisions of JGT 408-2019, the strength of grouting material should not be less than 85MPa. Is the strength in this article too low? (Table 4)**

**Response:** Thank you for the expert comments. The load transfer mechanism is different: the connector in this paper relies on the cooperative load transfer of the stud end bearing, steel tube confinement, and local bearing of the grout, rather than the bond anchorage between the rebar and the grout. Experimental results show that grout with a strength of 44–64 MPa is already sufficient to allow the stud to fully yield and fracture, and the grout strength meets the design requirements, with no grout crushing failure observed. Purpose of the parametric study: This paper adopts low-to-medium strength grout, aiming to investigate the ultimate load-bearing potential and failure mechanism of the connector under low-strength grout. This provides a reference for the selection of low-cost and easy-to-construct grout materials in engineering practice, and falls within the scope of fundamental research rather than direct design for engineering applications. Furthermore, the numerical simulation analysis also demonstrates the effectiveness of using low-strength grout in the connector.

**8. Please draw a diagram to illustrate the loading procedure in Section 2.4.**

**Response:** Thank you for the expert comments. We have added two schematic diagrams in Section 2.4 to clearly illustrate the detailed loading procedure in the revised manuscript. As shown in the figure below:

|  |  |
| --- | --- |
| (a) Schematic diagram | (b) Photograph of actual equipment |
| **Fig. 8.** Test loading setup. | |

**9. “The initial stiffness K, yield load Py, ultimate load Pu, maximum deformation Δmax”, Please explain in detail how these values were obtained and calculated? (Section 3.1)**

**Response:** Thank the expert for the comments. Initial stiffness K: The tangent stiffness at the point corresponding to one-third of the ultimate load. Yield load Py: Determined using the 0.2% residual strain method. Ultimate load Pu: The peak load on the load–deformation curve (as indicated in Figure 10). Maximum deformation Δmax: The deformation corresponding to the peak load on the load–deformation curve.

**10. It is recommended to keep two decimal places. (Table 5)**

**Response:** Thank the expert for the comments. We agree that maintaining an appropriate level of precision improves the clarity and professionalism of the table. Therefore, we have revised Table 5, and all numerical data are now presented to two decimal places. The relevant changes have been made in the revised manuscript.

**11. It is suggested to add a discussion chapter to summarize and analyze the influence of research parameters on connection performance.**

**Response:** Thank the expert for the comments. We have added a new section, Section 3.7, to summarize and analyze the influence of the research parameters (stud embedment depth, steel tube thickness, base plate hole diameter in the steel tube, stud type, and stud end diameter) on connection performance. The relevant revisions have been made in the revised manuscript.

**12. Please draw a diagram to describe the constitutive relationship of the material and indicate the specific values in the diagram. (Section 4.1)**

**Response:** Thank the expert for the comments. We have added the schematic diagrams and table to describe the material constitutive relationship in Section 4.1, and marked the corresponding specific key values on the diagram as required.

|  |  |
| --- | --- |
| **Fig.18.** Stress-strain curve of steel | **Fig.19.** Concrete constitutive model |

**Table 6.** CDP model parameters of the grout material

| Dilation Angle *Ψ*/° | Eccentricity *ε* | fb0/fc0 *α_f_* | K | Viscosity Parameter *µ* |
| --- | --- | --- | --- | --- |
| 30 | 0.1 | 1.16 | 2/3 | 0.0005 |

**13. The author should highlight the practical value of the research in the introduction.**

**Response:** Thank the expert for the comments. We have supplemented and highlighted the practical engineering value and application significance of this research in the Introduction section.

**Reviewer #2: The manuscript titled “Research on the anti-pull performance of a novel small-diameter steel tube-stud grouting connector” addresses a pertinent research topic within the realm of structural engineering. The presented study refers to a study that investigates how well a newly designed connector resists being pulled apart by tensile forces. The connector is “novel” because it is a new type, made from a small-diameter steel tube that contains a short steel rod (stud), with the space between them filled by high-strength grout. When the grout hardens, it bonds the stud to the tube and to the surrounding structural element, allowing forces to be safely transferred. The term “anti-pull performance” describes the connector’s ability to resist pull-out, often evaluated in terms of ultimate pull-out strength, load–displacement behavior, and failure modes such as grout cracking or bond failure. This kind of research is important for applications like prefabricated concrete structures and steel–concrete composite systems, where compact, reliable connectors are needed to ensure that elements remain securely joined under tensile loading. To enhance the manuscript's clarity, it is essential to refine the articulation of key concepts, ensuring that the arguments are logically structured and effectively communicated. Additionally, the methodological approach should be strengthened by providing a more detailed explanation of the research design, data analysis techniques, and validation processes to ensure scientific rigor. Furthermore, a deeper discussion of the findings, including their broader implications and potential applications, would significantly enhance the manuscript’s impact. Addressing these aspects will improve the overall coherence, credibility, and contribution of the study, aligning it with the expected scholarly standards.**

**1. The abstract succinctly summarizes the key findings, emphasizing the influence of STG. This clarity is appreciated, but further elaboration on the significance of these findings would enhance the abstract's impact.**

**Response:** Thank the expert for the comments. We have further elaborated the research significance and application value of the key findings in the abstract to strengthen their academic impact. The significance of this research and its engineering application value are summarized as follows. This study fills an existing research gap regarding external-wall connections for steel structures and proposes an innovative, reliable connecting detail for prefabricated steel facade joints. Test results confirm that the proposed connection exhibits favourable pullout behaviour even with low-strength grout and a shallow stud embedment of 4d, which reduces material costs and simplifies field construction. The derived formula for yield load capacity achieves high prediction accuracy (The error is less than 11.3%) and applies to practical engineering calculations, thereby enhancing design efficiency and structural safety. In addition, this work provides theoretical underpinnings and experimental datasets for parametric optimization, engineering popularization, and the development of specifications for STG connectors.

**2. The selection of keywords appears inadequate, as they fail to comprehensively encompass all facets of the research and accurately communicate the underlying concepts. A revision is recommended to ensure a more precise representation of the study’s scope and content.**

**Response:** Thank the expert for the comments. We have carefully reviewed and revised the keywords, supplemented appropriate terms, and optimized the selection to fully and accurately cover the research scope, core concepts, and main content of this study. The revised keywords are: Steel tube-stud grouting connector between steel frame and external wall; Pull-out performance; Finite element analysis; Mohr-Coulomb strength theory; Yield bearing capacity.

**3. The introduction needs a clearer structure and flow. The information needs to be more -cohesive, making it easier for the reader to follow the logical progression of ideas.**

**Response:** Thank the expert for the comments. We have reorganised the structure and logical flow of the introduction, improved the cohesion between paragraphs, and optimized the logical progression for better readability.

**4. The introduction does not provide a clear conclusion or summary of the information presented. It is important to provide a concise summary of the key points discussed in the introduction.**

**Response:** Thank the expert for the comments. We have added a concise concluding summary at the end of the introduction to summarize the key points and complete the logical arrangement of this section.

**5. The introduction section needs to expand. The literature review seems very limited.**

**Response:** Thank the expert for the comments. We have expanded the content of the introduction and supplemented more relevant references to enrich the literature review section.

**6. The authors should provide a more explicit delineation of the inherent novelty in their research. It is imperative that they articulate the innovative aspects of their study and specify any prerequisites essential for its execution. This comparative analysis would enhance the clarity and depth of understanding regarding the distinct contributions of the current research in relation to these seminal works in the field. I suggest to add the section of research significance.**

**Response:** Thank the expert for the comments. We have clearly clarified the inherent novelty and innovative aspects of this research, added the necessary prerequisites for implementation, and conducted a comparative analysis with previous seminal studies to highlight our distinct contributions. Meanwhile, we have supplemented the section on research significance as suggested.

**7. Highlighting the novelty and significance of research findings within the introduction would further engage readers.**

**Response:** Thank the expert for the comments. This paper presents experimental, numerical, and theoretical investigations into the pullout behaviour of steel tube‑stud grouted connections. The novelties of this work are threefold: (1) identification of a synergistic load-transfer mechanism combining stud end bearing, steel tube confinement, and localized grout support, which differs from the conventional bond and anchorage mechanism of reinforced-bar grouted joints. (2) assessment of low-to-medium-strength grouts and specimens with shallow stud embedment, demonstrating that such connections can maintain satisfactory mechanical performance. (3) quantification of the effects of critical parameters (embedment depth, tube wall thickness, etc.) on failure modes and ultimate pullout capacity. This research provides valuable guidance for the future design and engineering application of tubular grouted connectors linking primary steel frames to external claddings, and offers benchmark data for finite-element modelling of comparable grouted connections. Once again, we thank the reviewers for their guidance. The above revisions have been made in the revised manuscript.

**8. The experimental phase of study was not presented suitable. It should improve.**

**Response:** Thank the expert for the comments. We have revised the descriptions of the experimental program (Chapter 2) to make them clearer and more complete, and all modifications have been highlighted in the revised manuscript.

**9. The regulations and standards of the tests was not presented. All of the regulations for each experiment should provide.**

**Response:** Thank the expert for the comments. We have now explicitly provided the relevant regulations and standards for each experiment in the revised manuscript (Sections 2.2.1,~2.4.1). The added standards include GB/T 228.1-2021 for steel tensile tests and GB/T 50448-2015 for grout tests. The changes are highlighted in the revised manuscript.

**10. More comparative analysis with experimental and other studies in the technical literature should be done.**

**Response:** Thank the expert for the comments. Owing to the relatively novel configuration of the proposed STG connector (stud end bearing + steel tube confinement + local grout bearing), few directly comparable experimental data have been reported in the literature. We will continue to monitor relevant published results and conduct comprehensive comparative analyses in future work once sufficient test data become available. We appreciate the reviewer’s understanding and constructive comments.

**11. The conclusion section could benefit from more contextual information about the significance of the research findings. It is helpful to know how the research findings could be applied in real-world situations or how they contribute to existing knowledge in the field.**

**Response:** Thank the expert for the comments. We have supplemented the research significance in the conclusion section and further illustrated the practical application prospects of the findings, as well as the academic contributions to the existing knowledge in this field. The main contributions and research significance of this paper are as follows: (1) The proposed connector, featuring low-cost, short embedment depth, and low-strength grout, can substantially cut material expenses, simplify construction procedures, improve site assembly efficiency, and satisfy the demands of industrialized construction. (2) Existing analytical theories for grouted connections primarily focus on rebar sleeve connections and lack dedicated mechanical models for the composite system consisting of steel tube confinement and stud-end bearing. In this work, a formula for yield bearing capacity is derived based on the Mohr–Coulomb strength criterion and elastic confinement mechanism, with calculation errors within 11.3%. This finding bridges the theoretical gap for steel-structural grouted connections and complements the analytical framework of such connection systems. (3) This study verifies that satisfactory pullout performance can be realized with medium–low strength grout, breaking the conventional design mindset of adopting high-grade grout and long embedment lengths. The research further clarifies the synergistic load-transfer mechanism among steel tube confinement, stud end bearing, and the localized grout crushing, supplying essential test data and theoretical foundations for future parametric optimization and numerical simulation.

**12. Lots of references are outdated. Please expand them. If a suitable position is found, authors can cite references below. [1] Influence of repeated heating–cooling cycles and exposure duration on mechanical, electrical, and durability properties of geopolymer concrete. [2] Residual axial performance of PET/rubber-modified concrete confined with CFRP strips after thermal exposure: Experimental and theoretical analysis. [3] Thermo-mechanical behavior of high-strength concrete with nylon granule aggregates: Experimental evaluation and predictive analysis. [4] Effect of specimen size on compressive capacity and damage mechanisms of PET-containing self-consolidating concrete after high-temperature exposure. [5] Improvement of Recycled Concrete Aggregate Properties by Polyvinyl Alcohol. [6] Evaluation of the Static Behavior of WPC-GFRP Sandwich Panels: An Experimental, Theoretical, and Numerical Study. [6] Improvement of Recycled Concrete Aggregate Properties by Polyvinyl Alcohol**

**Response:** Thank the expert for the comments. Most references suggested by the reviewer have been cited and placed in appropriate locations within the Introduction, and all modifications are highlighted in the revised manuscript. One reference is not incorporated, as its research topic lacks direct relevance to this work.

**13. No in-depth conclusion was found.**

**Response:** Thank the expert for the comments. We have enhanced the conclusion by adding the research value and practical engineering applications of our findings, and by clarifying how they complement and advance existing theories and research frameworks in the field. These revisions have been completed in the revised manuscript.

**14. Improve the quality of figures and charts. The quality if the presented figures are not suitable for publication.**

**Response:** Thank the expert for the comments. We have comprehensively improved the quality of all figures and charts, including enhancing the resolution, optimizing the clarity of lines, and adjusting the layout to meet the publication requirements.

**15. All the commercial names should be deleted.**

**Response:** Thank the expert for the comments. We have thoroughly reviewed the entire manuscript and removed all commercial information. Only generic material names, standard terminology, standard numbers, and academic expressions are retained. All commercial identifiers have been replaced with neutral, general technical descriptions. This ensures the paper complies with academic norms and contains no commercial promotion.

**16. Check the article grammatically.**

**Response:** Thank the expert for the comments. We have carefully checked and revised the whole manuscript for grammatical errors and polished the language expression throughout the article.

**17. All the citations in the manuscript need to be double-checked to ensure their consistency with the reference list.**

**Response:** Thank the expert for the comments. We have carefully double-checked all in-text citations and fully unified them with the reference list in the revised manuscript.

**18. Remove the gray border of the presented curves.**

**Response:** Thank the expert for the comments. We have removed the gray border of all curves in the revised figures.
