# Supplementary material for: Research on the anti-pull performance of a novel small-diameter steel tube-stud grouting connector
Source: PLoS One. 2026 Jul 22;21(7):e0353244. doi: 10.1371/journal.pone.0353244 (PMC13390846; doi:10.1371/journal.pone.0353244)
Supplement: Peer review history

# Peer review: Decision Letter 0

**DOI:** 10.1371/journal.pone.0353244.r001

**Authors:** Zhenhua Li

27 Apr 2026

PONE-D-26-15500

Research on the anti-pull performance of a novel small-diameter steel tube-stud grouting connector

PLOS One

Dear Dr. Xiao,

Thank you for submitting your manuscript to PLOS ONE. After careful consideration, we feel that it has merit but does not fully meet PLOS ONE’s publication criteria as it currently stands. Therefore, we invite you to submit a revised version of the manuscript that addresses the points raised during the review process.

**You will see from the referees' comments that additional information needs to be provided, and we ask that this be provided, before we consider you manuscript further.**

Please submit your revised manuscript by Jun 11 2026 11:59PM. If you will need more time than this to complete your revisions, please reply to this message or contact the journal office at plosone@plos.org. When you're ready to submit your revision, log on to https://www.editorialmanager.com/pone/ and select the 'Submissions Needing Revision' folder to locate your manuscript file.

Please include the following items when submitting your revised manuscript:

- A letter that responds to each point raised by the academic editor and reviewer(s). You should upload this letter as a separate file labeled 'Response to Reviewers'.
- A marked-up copy of your manuscript that highlights changes made to the original version. You should upload this as a separate file labeled 'Revised Manuscript with Track Changes'.
- An unmarked version of your revised paper without tracked changes. You should upload this as a separate file labeled 'Manuscript'.

If you would like to make changes to your financial disclosure, please include your updated statement in your cover letter. Guidelines for resubmitting your figure files are available below the reviewer comments at the end of this letter.

If applicable, we recommend that you deposit your laboratory protocols in protocols.io to enhance the reproducibility of your results. Protocols.io assigns your protocol its own identifier (DOI) so that it can be cited independently in the future. For instructions see: https://journals.plos.org/plosone/s/submission-guidelines#loc-laboratory-protocols. Additionally, PLOS ONE offers an option for publishing peer-reviewed Lab Protocol articles, which describe protocols hosted on protocols.io. Read more information on sharing protocols at https://plos.org/protocols?utm_medium=editorial-email&utm_source=authorletters&utm_campaign=protocols.

As the corresponding author, your ORCID iD is verified in the submission system and will appear in the published article. PLOS supports the use of ORCID, and we encourage all coauthors to register for an ORCID iD and use it as well. Please encourage your coauthors to verify their ORCID iD within the submission system before final acceptance, as unverified ORCID iDs will not appear in the published article. *Only*  the individual author can complete the verification step; PLOS staff *cannot*  verify ORCID iDs on behalf of authors.

We look forward to receiving your revised manuscript.

Kind regards,

Zhenhua Li

Academic Editor

PLOS One

**Journal Requirements:**

1. When submitting your revision, we need you to address these additional requirements.

Please ensure that your manuscript meets PLOS ONE's style requirements, including those for file naming. The PLOS ONE style templates can be found at

https://journals.plos.org/plosone/s/file?id=wjVg/PLOSOne_formatting_sample_main_body.pdf and

https://journals.plos.org/plosone/s/file?id=ba62/PLOSOne_formatting_sample_title_authors_affiliations.pdf

2. Please note that PLOS One has specific guidelines on code sharing for submissions in which author-generated code underpins the findings in the manuscript. In these cases, we expect all author-generated code to be made available without restrictions upon publication of the work. Please review our guidelines at https://journals.plos.org/plosone/s/materials-and-software-sharing#loc-sharing-code and ensure that your code is shared in a way that follows best practice and facilitates reproducibility and reuse.

3. We note that the grant information you provided in the ‘Funding Information’ and ‘Financial Disclosure’ sections do not match.

When you resubmit, please ensure that you provide the correct grant numbers for the awards you received for your study in the ‘Funding Information’ section.

4. Thank you for stating the following financial disclosure:

Guangxi Key Laboratory of New Energy and Building Energy Saving Project (Grant no. RZ2000000384).

Please state what role the funders took in the study.  If the funders had no role, please state: "The funders had no role in study design, data collection and analysis, decision to publish, or preparation of the manuscript."

If this statement is not correct you must amend it as needed.

Please include this amended Role of Funder statement in your cover letter; we will change the online submission form on your behalf.

5. Thank you for stating the following in the Acknowledgments Section of your manuscript:

This research was supported by the Talent Fund of Guizhou University (2021) No.10, and the Guangxi Key Laboratory of New Energy and Building Energy Saving Project (Grant No. RZ2000000384).

We note that you have provided funding information that is not currently declared in your Funding Statement. However, funding information should not appear in the Acknowledgments section or other areas of your manuscript. We will only publish funding information present in the Funding Statement section of the online submission form.

Please remove any funding-related text from the manuscript and let us know how you would like to update your Funding Statement. Currently, your Funding Statement reads as follows:

Guangxi Key Laboratory of New Energy and Building Energy Saving Project (Grant no. RZ2000000384).

Please include your amended statements within your cover letter; we will change the online submission form on your behalf.

6. When completing the data availability statement of the submission form, you indicated that you will make your data available on acceptance. We strongly recommend all authors decide on a data sharing plan before acceptance, as the process can be lengthy and hold up publication timelines. Please note that, though access restrictions are acceptable now, your entire data will need to be made freely accessible if your manuscript is accepted for publication. This policy applies to all data except where public deposition would breach compliance with the protocol approved by your research ethics board. If you are unable to adhere to our open data policy, please kindly revise your statement to explain your reasoning and we will seek the editor's input on an exemption. Please be assured that, once you have provided your new statement, the assessment of your exemption will not hold up the peer review process.

7. PLOS requires an ORCID iD for the corresponding author in Editorial Manager on papers submitted after December 6th, 2016. Please ensure that you have an ORCID iD and that it is validated in Editorial Manager. To do this, go to ‘Update my Information’ (in the upper left-hand corner of the main menu), and click on the Fetch/Validate link next to the ORCID field. This will take you to the ORCID site and allow you to create a new iD or authenticate a pre-existing iD in Editorial Manager.

8. We note that Figures 5, 6, 11, 12, 13 and 16 in your submission contain copyrighted images. All PLOS content is published under the Creative Commons Attribution License (CC BY 4.0), which means that the manuscript, images, and Supporting Information files will be freely available online, and any third party is permitted to access, download, copy, distribute, and use these materials in any way, even commercially, with proper attribution. For more information, see our copyright guidelines: http://journals.plos.org/plosone/s/licenses-and-copyright.

We require you to either present written permission from the copyright holder to publish these figures specifically under the CC BY 4.0 license, or remove the figures from your submission:

a. You may seek permission from the original copyright holder of Figures 5, 6, 11, 12, 13 and 16 to publish the content specifically under the CC BY 4.0 license.

We recommend that you contact the original copyright holder with the Content Permission Form (http://journals.plos.org/plosone/s/file?id=7c09/content-permission-form.pdf) and the following text:

“I request permission for the open-access journal PLOS ONE to publish XXX under the Creative Commons Attribution License (CCAL) CC BY 4.0 (http://creativecommons.org/licenses/by/4.0/). Please be aware that this license allows unrestricted use and distribution, even commercially, by third parties. Please reply and provide explicit written permission to publish XXX under a CC BY license and complete the attached form.”

Please upload the completed Content Permission Form or other proof of granted permissions as an "Other" file with your submission.

In the figure caption of the copyrighted figure, please include the following text: “Reprinted from [ref] under a CC BY license, with permission from [name of publisher], original copyright [original copyright year].”

b. If you are unable to obtain permission from the original copyright holder to publish these figures under the CC BY 4.0 license or if the copyright holder’s requirements are incompatible with the CC BY 4.0 license, please either i) remove the figure or ii) supply a replacement figure that complies with the CC BY 4.0 license. Please check copyright information on all replacement figures and update the figure caption with source information. If applicable, please specify in the figure caption text when a figure is similar but not identical to the original image and is therefore for illustrative purposes only.

9. If the reviewer comments include a recommendation to cite specific previously published works, please review and evaluate these publications to determine whether they are relevant and should be cited. There is no requirement to cite these works unless the editor has indicated otherwise.

**Additional Editor Comments:**

You will see from the referees' comments that additional information needs to be provided, and we ask that this be provided, before we consider you manuscript further.

[Note: HTML markup is below. Please do not edit.]

Reviewers' comments:

Reviewer's Responses to Questions

**Comments to the Author**

1. Is the manuscript technically sound, and do the data support the conclusions?

The manuscript must describe a technically sound piece of scientific research with data that supports the conclusions. Experiments must have been conducted rigorously, with appropriate controls, replication, and sample sizes. The conclusions must be drawn appropriately based on the data presented.

Reviewer #1: Partly

Reviewer #2: Yes

**********

2. Has the statistical analysis been performed appropriately and rigorously?

Reviewer #1: Yes

Reviewer #2: Yes

**********

3. Have the authors made all data underlying the findings in their manuscript fully available?

The PLOS Data policy requires authors to make all data underlying the findings described in their manuscript fully available without restriction, with rare exception (please refer to the Data Availability Statement in the manuscript PDF file). The data should be provided as part of the manuscript or its supporting information, or deposited to a public repository. For example, in addition to summary statistics, the data points behind means, medians and variance measures should be available. If there are restrictions on publicly sharing data—e.g. participant privacy or use of data from a third party—those must be specified.

Reviewer #1: Yes

Reviewer #2: Yes

**********

4. Is the manuscript presented in an intelligible fashion and written in standard English?

PLOS ONE does not copyedit accepted manuscripts, so the language in submitted articles must be clear, correct, and unambiguous. Any typographical or grammatical errors should be corrected at revision, so please note any specific errors here.

Reviewer #1: Yes

Reviewer #2: Yes

**********

5. Review Comments to the Author

Please use the space provided to explain your answers to the questions above. You may also include additional comments for the author, including concerns about dual publication, research ethics, or publication ethics. (Please upload your review as an attachment if it exceeds 20,000 characters)

**Reviewer #1:** General Comments:

This manuscript investigates the pull-out performance of steel tube–stud grouting connectors, with research parameters including embedment depth, stud steel type, steel tube thickness, stud end diameter, and the diameter of the backing plate hole. Based on the Mohr–Coulomb strength criterion and elastic theory, an analytical expression for the yield bearing capacity of connectors under tensile pull-out is developed by modeling the stress state of the surrounding mortar.

However, the manuscript is poorly organized, and numerous issues need to be addressed before it can be considered for publication.

Specific Comments:

1. The key words fail to present the main content of this article. It is suggested to replace the first key word.

2. There is no head diameter in Figure 4. The expression in Figure 4 should correspond to that in Table 1.

3. Have duplicate specimens been set up, with only one specimen of each type. How can accidental errors be avoided? (Section 2.1)

4. The diameter (d) of the stud should be supplemented in Table 1.

5. The diameter of the steel pipe should be indicated in Table 1.

6. Pictures of the tensile tests of stud bolts and steel pipes should be supplemented in Section 2.2.

7. According to the provisions of JGT 408-2019, the strength of grouting material should not be less than 85MPa. Is the strength in this article too low? (Table 4)

8. Please draw a diagram to illustrate the loading procedure in Section 2.4.

9. “The initial stiffness K, yield load Py, ultimate load Pu, maximum deformation Δmax”, Please explain in detail how these values were obtained and calculated? (Section 3.1)

10. It is recommended to keep two decimal places. (Table 5)

11. It is suggested to add a discussion chapter to summarize and analyze the influence of research parameters on connection performance.

12. Please draw a diagram to describe the constitutive relationship of the material and indicate the specific values in the diagram. (Section 4.1)

13. The author should highlight the practical value of the research in the introduction.

**Reviewer #2:** The manuscript titled “Research on the anti-pull performance of a novel small-diameter steel tube-stud grouting connector” addresses a pertinent research topic within the realm of structural engineering. The presented study refers to a study that investigates how well a newly designed connector resists being pulled apart by tensile forces. The connector is “novel” because it is a new type, made from a small-diameter steel tube that contains a short steel rod (stud), with the space between them filled by high-strength grout. When the grout hardens, it bonds the stud to the tube and to the surrounding structural element, allowing forces to be safely transferred. The term “anti-pull performance” describes the connector’s ability to resist pull-out, often evaluated in terms of ultimate pull-out strength, load–displacement behavior, and failure modes such as grout cracking or bond failure. This kind of research is important for applications like prefabricated concrete structures and steel–concrete composite systems, where compact, reliable connectors are needed to ensure that elements remain securely joined under tensile loading. To enhance the manuscript's clarity, it is essential to refine the articulation of key concepts, ensuring that the arguments are logically structured and effectively communicated. Additionally, the methodological approach should be strengthened by providing a more detailed explanation of the research design, data analysis techniques, and validation processes to ensure scientific rigor. Furthermore, a deeper discussion of the findings, including their broader implications and potential applications, would significantly enhance the manuscript’s impact. Addressing these aspects will improve the overall coherence, credibility, and contribution of the study, aligning it with the expected scholarly standards.

1- The abstract succinctly summarizes the key findings, emphasizing the influence of STG. This clarity is appreciated, but further elaboration on the significance of these findings would enhance the abstract's impact.

2- The selection of keywords appears inadequate, as they fail to comprehensively encompass all facets of the research and accurately communicate the underlying concepts. A revision is recommended to ensure a more precise representation of the study’s scope and content.

3- The introduction needs a clearer structure and flow. The information needs to be more -cohesive, making it easier for the reader to follow the logical progression of ideas.

4- The introduction does not provide a clear conclusion or summary of the information presented. It is important to provide a concise summary of the key points discussed in the introduction.

5- The introduction section needs to expand. The literature review seems very limited.

6- The authors should provide a more explicit delineation of the inherent novelty in their research. It is imperative that they articulate the innovative aspects of their study and specify any prerequisites essential for its execution. This comparative analysis would enhance the clarity and depth of understanding regarding the distinct contributions of the current research in relation to these seminal works in the field. I suggest to add the section of research significance.

7- Highlighting the novelty and significance of research findings within the introduction would further engage readers.

9- The experimental phase of study was not presented suitable. It should improve.

10- The regulations and standards of the tests was not presented. All of the regulations for each experiment should provide.

11- More comparative analysis with experimental and other studies in the technical literature should be done.

12- The conclusion section could benefit from more contextual information about the significance of the research findings. It is helpful to know how the research findings could be applied in real-world situations or how they contribute to existing knowledge in the field.

13- Lots of references are outdated. Please expand them. If a suitable position is found, authors can cite references below. [1] Influence of repeated heating–cooling cycles and exposure duration on mechanical, electrical, and durability properties of geopolymer concrete. [2] Residual axial performance of PET/rubber-modified concrete confined with CFRP strips after thermal exposure: Experimental and theoretical analysis. [3] Thermo-mechanical behavior of high-strength concrete with nylon granule aggregates: Experimental evaluation and predictive analysis. [4] Effect of specimen size on compressive capacity and damage mechanisms of PET-containing self-consolidating concrete after high-temperature exposure. [5] Improvement of Recycled Concrete Aggregate Properties by Polyvinyl Alcohol. [6] Evaluation of the Static Behavior of WPC-GFRP Sandwich Panels: An Experimental, Theoretical, and Numerical Study. [6] Improvement of Recycled Concrete Aggregate Properties by Polyvinyl Alcohol

14- No in-depth conclusion was found.

15- Improve the quality of figures and charts. The quality if the presented figures are not suitable for publication.

16- All the commercial names should be deleted.

17- Check the article grammatically.

18- All the citations in the manuscript need to be double-checked to ensure their consistency with the reference list.

19- Remove the gray border of the presented curves.

**********

6. PLOS authors have the option to publish the peer review history of their article (what does this mean?). If published, this will include your full peer review and any attached files.

If you choose “no”, your identity will remain anonymous but your review may still be made public.

**Do you want your identity to be public for this peer review?**  For information about this choice, including consent withdrawal, please see our Privacy Policy.

Reviewer #1: No

Reviewer #2: No

**********

[NOTE: If reviewer comments were submitted as an attachment file, they will be attached to this email and accessible via the submission site. Please log into your account, locate the manuscript record, and check for the action link "View Attachments". If this link does not appear, there are no attachment files.]

To ensure your figures meet our technical requirements, please review our figure guidelines: https://journals.plos.org/plosone/s/figures

You may also use PLOS’s free figure tool, NAAS, to help you prepare publication quality figures: https://journals.plos.org/plosone/s/figures#loc-tools-for-figure-preparation.

NAAS will assess whether your figures meet our technical requirements by comparing each figure against our figure specifications.

# Author comment: Author response to Decision Letter 1

**DOI:** 10.1371/journal.pone.0353244.r002

10 Jun 2026

Reviewer #1: General Comments:

This manuscript investigates the pull-out performance of steel tube–stud grouting connectors, with research parameters including embedment depth, stud steel type, steel tube thickness, stud end diameter, and the diameter of the backing plate hole. Based on the Mohr–Coulomb strength criterion and elastic theory, an analytical expression for the yield bearing capacity of connectors under tensile pull-out is developed by modeling the stress state of the surrounding mortar. However, the manuscript is poorly organized, and numerous issues need to be addressed before it can be considered for publication.

1. The key words fail to present the main content of this article. It is suggested to replace the first key word.

Response: Thank the expert for the comments. We replaced the first keyword with a more appropriate one and added a keyword. The revised keywords accurately reflect the main content of the revised article, as shown below. The revised keywords: Steel tube-stud grouting connector between steel frame and external wall; Pull-out performance; Finite element analysis; Mohr-Coulomb strength theory; Yield bearing capacity

2. There is no head diameter in Figure 4. The expression in Figure 4 should correspond to that in Table 1.

Response: Thank the expert for the comments. We have supplemented the parameter of head diameter in Figure 4 and unified the symbol and expression format of Figure 4 to be consistent with Table 1 in the revised manuscript.

Fig. 4. Geometric structure diagram of the specimen

3. Have duplicate specimens been set up, with only one specimen of each type. How can accidental errors be avoided? (Section 2.1)

Response: Thank the expert for the comments. In this study, a total of 18 specimens were designed and divided into 5 groups (A, B, C, D, E) for parametric comparison. Multiple parallel specimens were arranged within each group. Key parameters (stud embedment depth, steel pipe wall thickness, base plate hole diameter in the steel pipe, stud type, and stud end diameter) each included 2 to 5 parallel specimens, rather than a single-specimen design. All grouting materials and steel were from the same batch. The specimens and test blocks were cured under the same conditions and loaded on the same day to minimize material variability. All specimens were subjected to the same loading rate, preloading cycles, and loading until failure, thereby eliminating accidental errors caused by different loading procedures.

4. The diameter (d) of the stud should be supplemented in Table 1.

Response: Thank the expert for the comments. We have supplemented the diameter d of the stud in Table 1 in the revised manuscript.

5. The diameter of the steel pipe should be indicated in Table 1.

Response: Thank the expert for the comments. We have indicated and supplemented the diameter of the steel pipe in Table 1 in the revised manuscript.

6. Pictures of the tensile tests of stud bolts and steel pipes should be supplemented in Section 2.2.

Response: Thank the expert for the comments. Due to an oversight during the experimental recording process, high-definition physical photographs of the tensile tests on the studs and steel tubes were not retained at the earlier stage. However, the relevant mechanical property data (yield strength, tensile strength, elastic modulus, elongation at fracture, etc.) have been tested in strict accordance with the standard (GB/T 228.1-2021) and are fully presented in Tables 2 and 3. The data are authentic and reliable. To improve the content of the paper, we have supplemented schematic diagrams of the tensile tests on the studs and steel pipes, along with dimensional annotations of the specimen geometry, in Section 2.2. Detailed written descriptions of the test procedure, loading method, and data acquisition method have also been provided, clearly presenting the entire process of material mechanical property testing and meeting the traceability requirements of the experiments. In future studies, we will strictly standardize the recording of experimental images to avoid similar oversights. Once again, we thank the reviewers for their guidance. The above revisions have been made in the revised manuscript.

(a) Schematic diagram of the specimen

(b) Loading diagram of the stud test

Fig. 5. Stud tensile test

(a) Schematic diagram of the specimen (b) Loading diagram of the steel tube test

Fig. 6. Steel tube tensile test

7. According to the provisions of JGT 408-2019, the strength of grouting material should not be less than 85MPa. Is the strength in this article too low? (Table 4)

Response: Thank you for the expert comments. The load transfer mechanism is different: the connector in this paper relies on the cooperative load transfer of the stud end bearing, steel pipe confinement, and local bearing of the grout, rather than the bond anchorage between the rebar and the grout. Experimental results show that grout with a strength of 44–64 MPa is already sufficient to allow the stud to fully yield and fracture, and the grout strength meets the design requirements, with no grout crushing failure observed. Purpose of the parametric study: This paper adopts low-to-medium strength grout, aiming to investigate the ultimate load-bearing potential and failure mechanism of the connector under low-strength grout. This provides a reference for the selection of low-cost and easy-to-construct grout materials in engineering practice, and falls within the scope of fundamental research rather than direct design for engineering applications. Furthermore, the numerical simulation analysis also demonstrates the effectiveness of using low-strength grout in the connector.

8. Please draw a diagram to illustrate the loading procedure in Section 2.4.

Response: Thank you for the expert comments. We have added two schematic diagrams in Section 2.4 to clearly illustrate the detailed loading procedure in the revised manuscript. As shown in the figure below:

(a) Schematic diagram (b) Photograph of actual equipment

Fig. 8. Test loading setup.

9. “The initial stiffness K, yield load Py, ultimate load Pu, maximum deformation Δmax”, Please explain in detail how these values were obtained and calculated? (Section 3.1)

Response: Thank the expert for the comments. Initial stiffness K: The tangent stiffness at the point corresponding to one-third of the ultimate load. Yield load Py: Determined using the 0.2% residual strain method. Ultimate load Pu: The peak load on the load–deformation curve (as indicated in Figure 10). Maximum deformation Δmax: The deformation corresponding to the peak load on the load–deformation curve.

10. It is recommended to keep two decimal places. (Table 5)

Response: Thank the expert for the comments. We agree that maintaining an appropriate level of precision improves the clarity and professionalism of the table. Therefore, we have revised Table 5, and all numerical data are now presented to two decimal places. The relevant changes have been made in the revised manuscript.

11. It is suggested to add a discussion chapter to summarize and analyze the influence of research parameters on connection performance.

Response: Thank the expert for the comments. We have added a new section, Section 3.7, to summarize and analyze the influence of the research parameters (stud embedment depth, steel pipe thickness, base plate hole diameter in the steel pipe, stud type, and stud end diameter) on connection performance. The relevant revisions have been made in the revised manuscript.

12. Please draw a diagram to describe the constitutive relationship of the material and indicate the specific values in the diagram. (Section 4.1)

Response: Thank the expert for the comments. We have added the schematic diagrams and table to describe the material constitutive relationship in Section 4.1, and marked the corresponding specific key values on the diagram as required.

Fig.18. Stress-strain curve of steel Fig.19. Concrete constitutive model

Table 6. CDP model parameters of the grout material

Dilation Angle Ψ/° Eccentricity ε fb0/fc0 αf K Viscosity Parameter µ

30 0.1 1.16 2/3 0.0005

13. The author should highlight the practical value of the research in the introduction.

Response: Thank the expert for the comments. We have supplemented and highlighted the practical engineering value and application significance of this research in the Introduction section.

Reviewer #2: The manuscript titled “Research on the anti-pull performance of a novel small-diameter steel tube-stud grouting connector” addresses a pertinent research topic within the realm of structural engineering. The presented study refers to a study that investigates how well a newly designed connector resists being pulled apart by tensile forces. The connector is “novel” because it is a new type, made from a small-diameter steel tube that contains a short steel rod (stud), with the space between them filled by high-strength grout. When the grout hardens, it bonds the stud to the tube and to the surrounding structural element, allowing forces to be safely transferred. The term “anti-pull performance” describes the connector’s ability to resist pull-out, often evaluated in terms of ultimate pull-out strength, load–displacement behavior, and failure modes such as grout cracking or bond failure. This kind of research is important for applications like prefabricated concrete structures and steel–concrete composite systems, where compact, reliable connectors are needed to ensure that elements remain securely joined under tensile loading. To enhance the manuscript's clarity, it is essential to refine the articulation of key concepts, ensuring that the arguments are logically structured and effectively communicated. Additionally, the methodological approach should be strengthened by providing a more detailed explanation of the research design, data analysis techniques, and validation processes to ensure scientific rigor. Furthermore, a deeper discussion of the findings, including their broader implications and potential applications, would significantly enhance the manuscript’s impact. Addressing these aspects will improve the overall coherence, credibility, and contribution of the study, aligning it with the expected scholarly standards.

1. The abstract succinctly summarizes the key findings, emphasizing the influence of STG. This clarity is appreciated, but further elaboration on the significance of these findings would enhance the abstract's impact.

Response: Thank the expert for the comments. We have further elaborated the research significance and application value of the key findings in the abstract to strengthen their academic impact. The significance of this research and its engineering application value are summarized as follows. This study fills an existing research gap regarding external-wall connections for steel structures and proposes an innovative, reliable connecting detail for prefabricated steel facade joints. Test results confirm that the proposed connection exhibits favourable pullout behaviour even with low-strength grout and a shallow stud embedment of 4d, which reduces material costs and simplifies field construction. The derived formula for yield load capacity achieves high prediction accuracy (The error is less than 11.3%) and applies to practical engineering calculations, thereby enhancing design efficiency and structural safety. In addition, this work provides theoretical underpinnings and experimental datasets for parametric optimization, engineering popularization, and the development of specifications for STG connectors.

2. The selection of keywords appears inadequate, as they fail to comprehensively encompass all facets of the research and accurately communicate the underlying concepts. A revision is recommended to ensure a more precise representation of the study’s scope and content.

Response: Thank the expert for the comments. We have carefully reviewed and revised the keywords, supplemented appropriate terms, and optimized the selection to fully and accurately cover the research scope, core concepts, and main content of this study. The revised keywords are: Steel tube-stud grouting connector between steel frame and external wall; Pull-out performance; Finite element analysis; Mohr-Coulomb strength theory; Yield bearing capacity.

3. The introduction needs a clearer structure and flow. The information needs to be more -cohesive, making it easier for the reader to follow the logical progression of ideas.

Response: Thank the expert for the comments. We have reorganised the structure and logical flow of the introduction, improved the cohesion between paragraphs, and optimized the logical progression for better readability.

4. The introduction does not provide a clear conclusion or summary of the information presented. It is important to provide a concise summary of the key points discussed in the introduction.

Response: Thank the expert for the comments. We have added a concise concluding summary at the end of the introduction to summarize the key points and complete the logical arrangement of this section.

5. The introduction section needs to expand. The literature review seems very limited.

Response: Thank the expert for the comments. We have expanded the content of the introduction and supplemented more relevant references to enrich the literature review section.

6. The authors should provide a more explicit delineation of the inherent novelty in their research. It is imperative that they articulate the innovative aspects of their study and specify any prerequisites essential for its execution. This comparative analysis would enhance the clarity and depth of understanding regarding the distinct contributions of the current research in relation to these seminal works in the field. I suggest to add the section of research significance.

Response: Thank the expert for the comments. We have clearly clarified the inherent novelty and innovative aspects of this research, added the necessary prerequisites for implementation, and conducted a comparative analysis with previous seminal studies to highlight our distinct contributions. Meanwhile, we have supplemented the section on research significance as suggested.

7. Highlighting the novelty and significance of research findings within the introduction would further engage readers.

Response: Thank the expert for the comments. This paper presents experimental, numerical, and theoretical investigations into the pullout behaviour of steel pipestud grouted connections. The novelties of this work are threefold: (1) identification of a synergistic load-transfer mechanism combining stud end bearing, steel pipe confinement, and localized grout support, which differs from the conventional bond and anchorage mechanism of reinforced-bar grouted joints. (2) assessment of low-to-medium-strength grouts and specimens with shallow stud embedment, demonstrating that such connections can maintain satisfactory mechanical performance. (3) quantification of the effects of critical parameters (embedment depth, pipe wall thickness, etc.) on failure modes and ultimate pullout capacity. This research provides valuable guidance for the future design and engineering application of tubular grouted connectors linking primary steel frames to external claddings, and offers benchmark data for finite-element modelling of comparable grouted connections. Once again, we thank the reviewers for their guidance. The above revisions have been made in the revised manuscript.

8. The experimental phase of study was not presented suitable. It should improve.

Response: Thank the expert for the comments. We have revised the descriptions of the experimental program (Chapter 2) to make them clearer and more complete, and all modifications have been highlighted in the revised manuscript.

9. The regulations and standards of the tests was not presented. All of the regulations for each experiment should provide.

Response: Thank the expert for the comments. We have now explicitly provided the relevant regulations and standards for each experiment in the revised manuscript (Sections 2.2.1,~2.4.1). The added standards include GB/T 228.1-20

Attachment Submitted filename: Response to Reviewers.docx

# Peer review: Decision Letter 1

**DOI:** 10.1371/journal.pone.0353244.r003

**Authors:** Zhenhua Li

21 Jun 2026

Research on the anti-pull performance of a novel small-diameter steel tube-stud grouting connector

PONE-D-26-15500R1

Dear Dr. Xiao,

We’re pleased to inform you that your manuscript has been judged scientifically suitable for publication and will be formally accepted for publication once it meets all outstanding technical requirements.

Within one week, you’ll receive an e-mail detailing the required amendments. When these have been addressed, you’ll receive a formal acceptance letter and your manuscript will be scheduled for publication.

An invoice will be generated when your article is formally accepted. Please note, if your institution has a publishing partnership with PLOS and your article meets the relevant criteria, all or part of your publication costs will be covered. Please make sure your user information is up-to-date by logging into Editorial Manager at Editorial Manager® and clicking the ‘Update My Information' link at the top of the page. For questions related to billing, please contact billing support.

If your institution or institutions have a press office, please notify them about your upcoming paper to help maximize its impact. If they’ll be preparing press materials, please inform our press team as soon as possible -- no later than 48 hours after receiving the formal acceptance. Your manuscript will remain under strict press embargo until 2 pm Eastern Time on the date of publication. For more information, please contact onepress@plos.org.

Kind regards,

Zhenhua Li

Academic Editor

PLOS One

Additional Editor Comments (optional):

accept

Reviewers' comments:

Reviewer's Responses to Questions

**Comments to the Author**

1. If the authors have adequately addressed your comments raised in a previous round of review and you feel that this manuscript is now acceptable for publication, you may indicate that here to bypass the “Comments to the Author” section, enter your conflict of interest statement in the “Confidential to Editor” section, and submit your "Accept" recommendation.

Reviewer #1: All comments have been addressed

Reviewer #2: All comments have been addressed

**********

2. Is the manuscript technically sound, and do the data support the conclusions?

The manuscript must describe a technically sound piece of scientific research with data that supports the conclusions. Experiments must have been conducted rigorously, with appropriate controls, replication, and sample sizes. The conclusions must be drawn appropriately based on the data presented.

Reviewer #1: Yes

Reviewer #2: Yes

**********

3. Has the statistical analysis been performed appropriately and rigorously?

Reviewer #1: Yes

Reviewer #2: Yes

**********

4. Have the authors made all data underlying the findings in their manuscript fully available?

The PLOS Data policy requires authors to make all data underlying the findings described in their manuscript fully available without restriction, with rare exception (please refer to the Data Availability Statement in the manuscript PDF file). The data should be provided as part of the manuscript or its supporting information, or deposited to a public repository. For example, in addition to summary statistics, the data points behind means, medians and variance measures should be available. If there are restrictions on publicly sharing data—e.g. participant privacy or use of data from a third party—those must be specified.

Reviewer #1: Yes

Reviewer #2: Yes

**********

5. Is the manuscript presented in an intelligible fashion and written in standard English?

PLOS ONE does not copyedit accepted manuscripts, so the language in submitted articles must be clear, correct, and unambiguous. Any typographical or grammatical errors should be corrected at revision, so please note any specific errors here.

Reviewer #1: Yes

Reviewer #2: Yes

**********

6. Review Comments to the Author

Please use the space provided to explain your answers to the questions above. You may also include additional comments for the author, including concerns about dual publication, research ethics, or publication ethics. (Please upload your review as an attachment if it exceeds 20,000 characters)

Reviewer #1: The authors have addressed the previous concerns and revised the manuscript as requested. I therefore recommend it for acceptance.

Reviewer #2: Comments have been addressed.

**********

7. PLOS authors have the option to publish the peer review history of their article (what does this mean?). If published, this will include your full peer review and any attached files.

If you choose “no”, your identity will remain anonymous but your review may still be made public.

**Do you want your identity to be public for this peer review?**  For information about this choice, including consent withdrawal, please see our Privacy Policy.

Reviewer #1: No

Reviewer #2: No

**********

# Editor report: Acceptance letter

**DOI:** 10.1371/journal.pone.0353244.r004

**Authors:** Zhenhua Li

PONE-D-26-15500R1

PLOS One

Dear Dr. Xiao,

I'm pleased to inform you that your manuscript has been deemed suitable for publication in PLOS One. Congratulations! Your manuscript is now being handed over to our production team.

At this stage, our production department will prepare your paper for publication. This includes ensuring the following:

* All references, tables, and figures are properly cited

* All relevant supporting information is included in the manuscript submission,

* There are no issues that prevent the paper from being properly typeset

You will receive further instructions from the production team, including instructions on how to review your proof when it is ready. Please keep in mind that we are working through a large volume of accepted articles, so please give us a few days to review your paper and let you know the next and final steps.

Lastly, if your institution or institutions have a press office, please let them know about your upcoming paper now to help maximize its impact. If they'll be preparing press materials, please inform our press team within the next 48 hours. Your manuscript will remain under strict press embargo until 2 pm Eastern Time on the date of publication. For more information, please contact onepress@plos.org.

You will receive an invoice from PLOS for your publication fee after your manuscript has reached the completed accept phase. If you receive an email requesting payment before acceptance or for any other service, this may be a phishing scheme. Learn how to identify phishing emails and protect your accounts at https://explore.plos.org/phishing.

If we can help with anything else, please email us at customercare@plos.org.

Thank you for submitting your work to PLOS ONE and supporting open access.

Kind regards,

PLOS ONE Editorial Office Staff

on behalf of

Professor Zhenhua Li

Academic Editor

PLOS One
